# The duplicated P450s *CYP6P9a*/*b* drive carbamates and pyrethroids cross-resistance in the major African malaria vector *Anopheles funestus*

**DOI:** 10.1371/journal.pgen.1010678

**Published:** 2023-03-27

**Authors:** Leon M. J. Mugenzi, Theofelix A. Tekoh, Sulaiman S. Ibrahim, Abdullahi Muhammad, Mersimine Kouamo, Murielle J. Wondji, Helen Irving, Jack Hearn, Charles S. Wondji

**Affiliations:** 1 LSTM Research Unit, Centre for Research in Infectious Diseases (CRID), Yaoundé, Cameroon; 2 Department of Biochemistry and Molecular Biology, Faculty of Science University of Buea, Buea, Cameroon; 3 Vector Biology Department, Liverpool School of Tropical Medicine, Pembroke Place, Liverpool, United Kingdom; 4 Department of Biochemistry, Bayero University, Kano, Nigeria; 5 Department of Biochemistry, Faculty of Science, University of Yaoundé 1, Yaoundé, Cameroon; 6 Centre for Epidemiology and Planetary Health, Department of Veterinary and Animal Science, North Faculty, Scotland’s Rural College, An Lòchran, 10 Inverness Campus, Inverness, Scotland, United Kingdom; London School of Hygiene & Tropical Medicine, UNITED KINGDOM

## Abstract

Cross-resistance to insecticides in multiple resistant malaria vectors is hampering resistance management. Understanding its underlying molecular basis is critical to implementation of suitable insecticide-based interventions. Here, we established that the tandemly duplicated cytochrome P450s, *CYP6P9a/b* are driving carbamate and pyrethroid cross-resistance in Southern African populations of the major malaria vector *Anopheles funestus*.

Transcriptome sequencing revealed that cytochrome P450s are the most over-expressed genes in bendiocarb and permethrin-resistant *An*. *funestus*. The *CYP6P9a* and *CYP6P9b* genes are overexpressed in resistant *An*. *funestus* from Southern Africa (Malawi) versus susceptible *An*. *funestus* (Fold change (FC) is 53.4 and 17 respectively), while the *CYP6P4a* and *CYP6P4b* genes are overexpressed in resistant *An*. *funestus* in Ghana, West Africa, (FC is 41.1 and 17.2 respectively). Other up-regulated genes in resistant *An*. *funestus* include several additional cytochrome P450s (e.g. *CYP9J5*, *CYP6P2*, *CYP6P5*), glutathione-S transferases, ATP-binding cassette transporters, digestive enzymes, microRNA and transcription factors (FC<7). Targeted enrichment sequencing strongly linked a known major pyrethroid resistance locus (*rp1*) to carbamate resistance centering around *CYP6P9a/b*. In bendiocarb resistant *An*. *funestus*, this locus exhibits a reduced nucleotide diversity, significant p-values when comparing allele frequencies, and the most non-synonymous substitutions. Recombinant enzyme metabolism assays showed that both *CYP6P9a/b* metabolize carbamates. Transgenic expression of *CYP6P9a/b* in *Drosophila melanogaster* revealed that flies expressing both genes were significantly more resistant to carbamates than controls. Furthermore, a strong correlation was observed between carbamate resistance and *CYP6P9a* genotypes with homozygote resistant *An*. *funestus* (*CYP6P9a* and the 6.5kb enhancer structural variant) exhibiting a greater ability to withstand bendiocarb/propoxur exposure than homozygote CYP6P9a_susceptible (e.g Odds ratio = 20.8, P<0.0001 for bendiocarb) and heterozygotes (OR = 9.7, P<0.0001). Double homozygote resistant genotype (RR/RR) were even more able to survive than any other genotype combination showing an additive effect.

This study highlights the risk that pyrethroid resistance escalation poses to the efficacy of other classes of insecticides. Available metabolic resistance DNA-based diagnostic assays should be used by control programs to monitor cross-resistance between insecticides before implementing new interventions.

## Author summary

Insecticide-based vector control measures are extensively used in the fight against malaria. Unfortunately, insecticides are in danger of losing their potency due to the development of resistance in vector species populations. The worst-case scenario is when a single resistance mechanism confers resistance to many insecticide classes, thereby hindering resistance management. The application of appropriate insecticide-based interventions depends critically on understanding the underlying molecular basis of insecticide resistance. Here, we performed gene expression profiling (RNAseq) and whole genome sequencing (PoolSeq) of multiple resistant *An*. *funestus* collected from Ghana, Uganda, Malawi, laboratory-resistant (FUMOZ), and susceptible (FANG) strains. We found overexpression of metabolic cytochrome P450s *CYP6P9a/b* genes in Malawi and *CYP6P4a/b* genes in Ghana and also detected signatures of a strong selection of some regions of the genome around these genes. *In vitro* expression of *CYP6P9a/b* P450s showed metabolic activities towards carbamates and transgenic flies overexpressing resistant alleles of both genes were significantly more resistant to carbamates. Furthermore, phenotype/genotype association studies revealed a strong correlation between carbamate resistance and *CYP6P9a/b*-resistant genotypes. Altogether, this study demonstrated that P450-based pyrethroid resistance also confers resistance to carbamate insecticides.

## Introduction

Malaria remains an important health burden in the tropical world with 95% of cases occurring in Africa [1]. In 2021, 247 million cases were reported worldwide with four countries (Nigeria, the Democratic Republic of the Congo, Uganda and Mozambique) accounting for almost half of all cases globally [1]. Vector control tools have been shown to be the most effective way of fighting malaria, credited with an estimated 517 million cases averted between 2000 and 2015 [2]. However, the effectiveness of these insecticide-based control tools is threatened by the emergence of insecticide resistance in major malaria vectors across African countries [3]. Indeed, the 2022 malaria report suggests that insecticide resistance is having an effect on epidemiological outcomes [1]. This link with malaria cases is further supported by the greater reduction in malaria burden in areas where bednets impregnated with pyrethroids and piperonyl butoxide (PBO) have been implemented [4,5]. PBO is a cytochrome P450-based metabolic resistance inhibitor which increases the potency of pyrethroids. Alarmingly, insecticide resistance in *Anopheles* mosquitoes continues to escalate and spread [6–10] in concert with the emergence of simultaneous resistance to several classes of insecticides, [7,11–14]. This multiplicity of resistance to insecticides from different classes further complicates the choice of suitable insecticides across Africa, especially for indoor residual spraying (IRS) limiting choices of resistance management strategies. Therefore, understanding the molecular drivers of cases of multi-resistance to different insecticide classes is critical to designing optimal control programs [15] as these cross-resistance mechanisms could also jeopardise current and future insecticides still under development. Such results will aid the design of insecticides that remain efficacious against the increasingly complex resistance patterns of local mosquitoes selected by both current insecticide-based interventions, but also agricultural pesticide use.

In the major malaria vector *An*. *funestus*, multiple resistance is increasingly documented, notably in Southern Africa and West Africa with resistance to both pyrethroids, carbamates and also DDT independently occurring in both regions [7,13,16,17]. Whereas in East and Central Africa, *An*. *funestus* is resistant to pyrethroids and DDT but not carbamates [9,18] [3,19,20]. Interestingly, all these populations have been shown to still remain susceptible to organophosphates [15]. Progress has been made in elucidating the molecular basis of pyrethroid resistance and that of DDT [21–26] with molecular markers detected for P450-based resistance including *CYP6P9a* [23], *CYP6P9b* [22] and for a 6.5kb structural variant insertion acting as an enhancer for both *CYP6P9a/b* expression [24]. These markers were subsequently used to design DNA-based diagnostic assays to track these specific instances of resistance by region across Africa [24]. However, DNA-based metabolic resistance markers have not yet been detected or associated with carbamate resistance in *An*. *funestus* although a cytochrome P450, *CYP6Z1* has previously been shown to metabolise this class of insecticide [27]. At present, a lack of such markers prevents fine-scale tracking of carbamate resistance.

Previous evidence of potential cross-resistance between pyrethroids and carbamates have been suggested in *An*. *funestus* with bioassay and synergist assays, and *CYP6Z1* was associated with such potential cross-resistance using *in vitro* heterologous assays [27]. However, it was not the most over-expressed metabolic resistance gene in Southern Africa or West Africa populations where pyrethroid and carbamate cross-resistance have been reported. This suggests that the major molecular drivers of such potential cross-resistance remain unidentified. Detecting the genes and variants driving resistance to carbamates or cross-resistance will surely shed light on the evolutionarily rapid adaptation of mosquitoes due to exposure to different insecticide classes which are currently in use. Furthermore, cross-resistance alleles of such genes can form molecular tests for potential cross-resistance between existing insecticides and novel ones currently in development.

This study is therefore aimed at characterizing the resistance profiles and elucidating the underlying molecular bases for carbamate resistance in *An*. *funestus*. Using RNAseq-based transcriptomic analysis and targeted-enrichment deep sequencing we detected the major genes associated with carbamate resistance and/or cross-resistance with pyrethroids in *An*. *funestus* in Africa. Through *in vitro* heterologous expression and *in vivo* assays, we demonstrated that the gene products of the duplicated cytochrome P450s *CYP6P9a* and *CYP6P9b* can metabolise carbamate and pyrethroid insecticides, conferring cross-resistance to these important public health insecticides. This was supported by strong correlations between genotypes of these genes and resistance to both insecticide classes. By establishing the known *CYP6P9a/b* markers as important tools to track cytochrome P450-based cross-resistance in field populations of vectors, our work will assist in the implementation of a more sophisticated management of resistance in malaria vectors.

## Materials and methods

### Collection and rearing of mosquitoes

Field mosquitoes used in this study were collected and reared between 2014 and 2021. Both laboratory and field samples of *An*. *funestus* were included in this study. The two laboratory strains were the FANG colony, a completely insecticide-susceptible colony originating from Angola, and the FUMOZ colony colonised from Southern Africa Mozambique. FUMOZ is pyrethroid and carbamates resistant [28], providing a good opportunity to study cross-resistance between both insecticide classes. *An*. *funestus* resistant to carbamates and pyrethroids were collected in Southern Africa [(Malawi (MWI), Chikwawa (16°1’ S, 34°47’ E) in 2014 [7])] and West [(Ghana (GHA), Obuasi (5°56′N, 1°37′W) in 2014 [13])]. Mosquitoes were also collected in eastern Africa (Uganda (UGA), Tororo (0°45’ N, 34°5’ E) in 2014 where populations are fully susceptible to carbamates but resistant to pyrethroids [18]. The collections were performed as previously described [23] by using electric aspirators to collect blood-fed, gravid indoor-resting females in houses between 06:00 AM-10.00 AM in each location. After allowing female mosquitoes to become fully gravid and the eggs to mature within 4 days post collection, they were introduced into 1.5ml Eppendorf tubes to lay eggs using the forced-egg laying protocol [29]. The F_1_ adults were generated after rearing larvae from field females and WHO bioassays were performed to assess the susceptibility patterns of each sample as previously described [23]. Morphological and molecular identifications were conducted to confirm the species as described previously [23].

### RNA extraction, library preparation and sequencing

Total RNA was extracted from three pools of 10 female *An*. *funestus*, for each sample group using the Arcturus PicoPure RNA isolation kit (Life Technologies, Carlsbad, CA, USA), according to the manufacturer’s instructions. Samples comprised *An*. *funestus* that survived exposure to 0.75% permethrin (MWI, UGA and GHA), 0.1% bendiocarb (MWI and GHA), 4% DDT (MWI and UGA), as well as control samples of *An*. *funestus* not exposed to any insecticide (MWI, UGA, GHA). Four replicates from the FANG (strain susceptible to all insecticides) and the FUMOZ (strain has multi-insecticide resistance) colonies were also extracted. All sequence library preparation, sequencing and quality trimming were performed by the Centre for Genomic Research (CGR), University of Liverpool. Pools of libraries (8/lane) were sequenced (2x125 bp paired-end sequencing) with v4 chemistry on a HiSeq 2500 (Illumina, San Diego, CA, USA).

### Analysis of RNAseq data

Analysis of the RNAseq data were performed as described for the transcriptional profiling of pyrethroid resistance in *An*. *funestus* populations Africa-wide [22,23], including the initial processing and quality assessment of the data. The analysis used the chromosome-scale *Anopheles funestus* FUMOZ colony reference genome assembly AfunF3 and annotation gene set AfunF3.1 [downloaded from https://www.vectorbase.org/ 25^th^ June 2019 [30]] [31]. To improve the functional annotation of the AfunF3.1 geneset, which contains many putative genes with no descriptions, Blast2Go [32] was used based on the non-redundant (nr) protein database downloaded from NCBI. Blast (BLASTx) searches against the non-redundant (nr) protein database and InterProScan searches of the InterPro protein signature databases were carried out to further annotate the *An*. *funestus* protein-coding genes.

The Strand NGS software, version 3.4 (Strand Life Sciences, Bangalore, India) was used for data analysis as previously described [22,23]. Further details are also presented in S1 Table (Tables A-C). Raw gene expression was quantified in Strand NGS and raw counts were normalised using DESeq’s inbuilt method [33] which accounts for differences in the total number of reads between samples.

Differential gene expression analysis was performed using DESeq [33] as implemented in Strand NGS. The estimated log_2_ fold change for each transcript was tested using a moderated t-test which is a modification of the unpaired t-test [34]. P-values were adjusted for multiple testing using the False Discovery Rate (FDR) approach of Storey with bootstrapping (q-value). Differentially expressed transcripts were defined as those with an FDR-adjusted P-value < 5% and fold-change > = 2. Gene ontology enrichment analysis was subsequently carried out on differentially expressed gene sets using Strand NGS.

#### Experimental design

We applied an hierarchical analytical approach where we started to compare the field resistant carbamate/pyrethroid *An*. *funestus* to the fully susceptible laboratory strain FANG using a triangular experimental design including *An*. *funestus* that survived exposure to bendiocarb (Rb), those unexposed (C) and the FANG fully susceptible (S). These were also compared to the *An*. *funestus* that survived exposure to permethrin (Rp). The assumption being that genes detected would be potentially associated with either resistance to one or both insecticide classes. In a second analysis, we also compared the field populations carbamate resistant to the field susceptible bendiocarb to better disentangle which genes are more associated to carbamate than pyrethroid resistance. This is because both of the field populations used for this particular analysis (Uganda and Malawi) are resistant to pyrethroids but with different genes over-expressed. For example *An*. *funestus* from Malawi (Southern Africa) exhibit a high overexpression of the P450 *CYP6P9a/b* genes whereas Ugandan (East Africa) over-expressed *CYP9K1* gene [35]. Therefore the best experimental approach is to compare which genes are overexpressed in carbamate/pyrethroid resistant *An*. *funestus* versus the laboratory colonized FANG strain (susceptible) as well as *An*. *funestus* field samples which are susceptible to carbamates. This would confirm which genes are linked with carbamate resistance.

Similarly, we used the FUMOZ laboratory strain because it is both resistant to carbamate and pyrethroids and has also originated from Southern Africa, we assumed that the molecular drivers of carbamate resistance or cross-resitance to pyrethroid would be further confirmed if also detect in FUMOZ.

For the comparison of field samples, twelve replicates of *An*. *funestus* from Malawi (3 permethrin alive, 3 bendiocarb alive, 3 DDT alive, 3 unexposed) were pooled with 4 replicates of *An*. *funestus* from the laboratory pyrethroid/bendiocarb resistant FUMOZ and considered as bendiocarb resistant. For bendiocarb susceptible, 9 replicates from Uganda (3 permethrin alive, 3 DDT alive, 3 unexposed) were combined with 4 replicates from FANG fully susceptible laboratory strain to form a susceptible bendiocarb sample irrespective of the geographical origin focusing only on the bendiocarb susceptibility status. This analysis was complementary to triangular design used in each country between 3 replicates of bendiocarb resistant (R), 3 replicates of unexposed (Control; C) and four replicates of the fully carbamate/pyrethroid susceptible FANG. The additional RNAseq data for permethrin [22, 23] and DDT resistant *An*. *funestus* [36] were previously described.

### Quantitative reverse transcriptase PCR

The expression patterns of the 12 most differentially expressed detoxification genes between different comparisons or countries were confirmed using qRT-PCR using already published primers and a previously described protocol (see Mugenzi et al. 2019 [22], S4 Table).

### SNP discovery or polymorphism based on Southern Africa/FUMOZ vs Uganda/FANG

#### Design of the sureselect

A detailed description of the sequence capture array is provided by Hearn et al. 2022 [35]. Briefly, the array was designed using a mix of *de novo* assembled *An*. *funestus* transcripts [37,38] and up-regulated genes in pyrethroid resistant populations [21,39]. The entire genomic regions of the major QTLs associated with pyrethroid resistance which are the 120kb BAC clone of the *rp1* containing the major CYP6 P450 cluster on the 2R chromosome arm as well as the 113kb BAC clone sequence for the *rp2* on the 2L chromosome arm were also included. A total of 1302 target sequences were included (with redundancy). More details about the bait design, library preparation and capture are provided in Supplementary Materials (S1 Text).

Initial processing and quality control of the targeted sequenced genomic regions were performed using StrandNGS 3.4 (Strand Life Sciences, Bangalore, India) following Hearn et al. 2022 [35]. Alignment and mapping were performed using the “DNA alignment” option against the recently annotated whole genome of *An*. *funestus* (version AfunF3.1) [31] (S1 Text). Aligned and mapped reads were used to create an experiment pipeline for DNA variant analysis.

The FUMOZ AfunF3.1 genome was used as the reference genome to detect all variant types [SNPs, MNPs (multiple nucleotide polymorphisms) and indels] using the MAQ independent model implemented in StrandNGS 3.4 and default parameters. A SNP multi sample report was generated for each sample. For each variant, its effect was predicted using the transcript annotation. To identify SNPs significantly associated with bendiocarb resistance, all *An*. *funestus* from Southern Africa (Malawi) and from the FUMOZ laboratory strain were grouped as these populations are known to be resistant to bendiocarb (totalling 38 *An*. *funestus*). The bendiocarb susceptible group was made of all *An*. *funestus* from Uganda known to be fully susceptible to bendiocarb (20) and those from the fully laboratory susceptible FANG strain (10). SNPs significantly associated with bendiocarb resistance were detected using two approaches. Firstly, we used a differential allele frequency-based approach where a variant was considered significantly associated with bendiocarb resistance if its supporting read range was between 1 and 25% in Southern African *An*. *funestus* (R). Such lower range is expected as Southern African *An*. *funestus* are similar to the FUMOZ reference genome. A higher range between 75 and 100% were expected for *An*. *funestus* from Uganda/FANG. A cut-off of supporting sample range of 19 out 38 for Southern Africa for Malawi/FUMOZ and 15 out of 30 (50% respectively) for Uganda/FANG was applied to select the significant SNPs. The second approach assessed the association between each variant and permethrin resistance by estimating the unpaired t-test of each variant between Southern Africa and Uganda/FANG, and a Manhattan plot of–Log_10_ of P-values were created.

#### Heterologous expression of recombinant *CYP6P9a and CYP6P9b* and metabolic assays *Cloning of full-length cDNA CYP6P9a and -b and protein expression*

Recombinant enzymes of *CYP6P9a* and *CYP6P9b* were expressed from the alleles cloned from Malawi *An*. *funestus* field samples (MAL-CYP6P9a and MAL-CYP6P9b), as previously described [40]. Membrane content and the P450 reductase activity were determined respectively as previously established [41,42].

### In vitro metabolism assays with bendiocarb

Metabolism assays were conducted with bendiocarb following protocols described previously [27]. The assay was carried out in 0.2 M Tris-HCl and NADPH-regeneration components added to the bottom of chilled 1.5 ml tubes. Membranes containing recombinant *CYP6P9a* or *-b* and *Ag*CPR were added to the side of the tube to which cytochrome b_5_ was already added in a ratio 1:4. These were pre-incubated for 5 min at 30°C, with shaking at 1,200 rpm, before adding 20 μM of bendiocarb, with continuous shaking at 1,200 rpm and 30°C for 2 h. Reactions were quenched with 0.1 ml ice-cold acetonitrile and incubated for 5 min to precipitate protein. Tubes were centrifuged at 16,000 rpm and 4°C for 15 min, and 150 μl of supernatant transferred into HPLC vials. 100 μl samples were injected into isocratic mobile phase (65:35 acetonitrile to water) with column temperature set to 40°C, wavelength of detection set to 205 nm and a flow rate of 1 ml/min. Peaks were separated with a 250 mm C18 column (Acclaim 120, Dionex) on an Agilent 1260 Infinity. All reactions were carried out in triplicate with experimental samples (+NADPH containing the NADPH regeneration buffer) and negative controls (-NADPH no NADPH regeneration buffer). Enzyme activity was calculated as percentage depletion (difference in the amount of bendiocarb remaining in the +NADPH tubes compared with the–NADPH) and a student’s t-test was used to estimate significance.

### In vivo transgenic expression of *An. funestus CYP6P9a/b* in Drosophila melanogaster flies

Transgenic *Drosophila melanogaster* expressing *CYP6P9a* or *CYP6P9b* genes were generated using the GAL4/UAS system to validate whether the over-expression of each of these genes alone can confer resistance to carbamates (bendiocarb). The same experimental setup had previously shown that over-expression of both genes confers resistance to type I (permethrin) and II (deltamethrin) pyrethroids [21,40]. The construction of the transgenic *D*. *melanogaster* strain was previously described [21,39]. Briefly, full-length *CYP6P9a and CYP6P9b* genes were amplified from cDNA using the Phusion High-Fidelity DNA Polymerase (Thermo Scientific) and cloned into the pJET1.2/blunt cloning vector (Thermo Scientific). For each gene, one predominant clone from Malawi was selected and cloned into the pUASattB vector using primers containing *Bgl*II and *Xba*I restriction sites. Using the PhiC31 system, the clones were injected into the germ-line of *D*. *melanogaster* w^1118^ strain. Two transgenic lines, UAS-CYP6P9a and UAS-CYP6P9b were obtained and balanced. A ubiquitous expression of each transgene in adult F_1_ progeny (experimental group) was obtained after crossing virgin females from the driver strain Act5C-GAL4 ["y [1] w [*]; P(Act5C-GAL4-w) E1/CyO","1;2"] (Bloomington Stock Centre, IN, USA) with UAS-CYP6P9a/b males. Similarly, adult F_1_ control progeny (control group) with the same genetic background as the experimental group but without *CYP6P9a or -b* insert were obtained by crossing virgin females from the driver strain Act5C-GAL4 and UAS recipient line males (which do not carry the pUASattB-CYP6P9a or -b insertion).

#### Bendiocarb susceptibility assays

A stock of 10 ml of 0.1% of insecticide was made by weighing 10 mg of the insecticide (0.1% bendiocarb (Sigma Aldrich)), then transferring into a 20 ml vial bottle containing 10 ml of acetone solvent (Sigma Aldrich). A 0.005% dilution of the stock was prepared using Silicone Dow oil. A second dilution of 0.007% was prepared to determine the best progress curve of mortality and knockdown of *Drosophila* flies to carbamate exposure. The impregnated papers were cut into two halves (6 cm x 15 cm), to fit the height of the fly bioassay plastic vials (45-cc plastic vials). The papers were rolled and inserted into the vial to cover the entire wall of the vial. Small cotton sugar balls (10% (wt/vol) sucrose) were inserted into each vial. A minimum of 20 to 25 flies were transferred into each vial. Mortalities were scored after 1, 2, 3, 4, 5, 6, 7, 8, 9, 10, 11, 12, and 24 h of exposure. Bioassay conditions were performed at 25 ± 5°C and 75 ± 10% relative humidity. Assays were performed in four replicates and Student’s t-test was used to compare the mortality plus knockdown between the experimental group and the control.

#### Confirmation of the overexpression of *CYP6P9a/b* genes by qPCR

To confirm the over-expression of *CYP6P9a/b* genes in the experimental flies and its absence in the control groups, qRT-PCR was performed as described previously [39,40]. First, RNA was extracted from three pools of 5 female flies from each fly group. cDNA (complimentary DNA) was synthesized using the extracted RNA, a set of three dilutions was done and dilution 2 was used to confirm the expression of *CYP6P9a* gene while being normalized by the house keeping gene ribosomal Protein (*RPL11*) [39]).

### Assessment of the correlation between *CYP6P9a and 6*.*5kb SV* genotypes and carbamate resistance

To assess the correlation between the genotypes of *CYP6P9a* resistant alleles and carbamate resistant phenotype, the FUMOZ-R and FANG-S *An*. *funestus* were crossed to obtain a hybrid line. The F_1_ progeny were then inter-crossed up to the fourth progeny (F_4_) after which carbamate bioassays (0.1% bendiocarb and 0.1% propoxur) were performed on adult females, aged 2–5 days [22,43]. DNA was then extracted from the legs of 40 alive and 40 dead female *An*. *funestus*, 24 hours after exposure. The resistant alleles were then genotyped in the alive and the dead *An*. *funestus* following the protocol previously designed [22,24].

### Statistical analysis

Data obtained from bioassays and genotyping was entered in an Excel spreadsheet where % mortality for bioassays and % genotypic frequencies and % allelic frequencies between the dead and alive group for genotyping were computed and bar plots created. GraphPad Prism statistical software (Version 9.0.2) was used to compute odds ratio (OR) showing the strength of association between genotype groups and ability to survive exposure and Fisher’s exact test at significance level *P = 0*.*05*, and 95% confidence interval (CI). The GraphPad Prism was also used to graphically illustrate bioassay, graphically illustrate the distribution of the various genotypes amongst alive and dead groups after exposure and qPCR results plot. Results were considered significant at *P*<0.05.

## Results

### Transcription profile of bendiocarb resistance

To detect the set of genes associated with bendiocarb resistance in Malawi, RNAseq was used to compare the bendiocarb resistant *An*. *funestus* (R) with control non-exposed *An*. *funestus* (C) (R vs. C comparison), and to the fully susceptible laboratory strain, FANG, S (R vs. S comparison). The control *An*. *funestus* were also compared to the susceptible FANG strain (C vs. S). A total of 466 transcripts were differentially expressed between R vs. S comparison with 187 up-regulated and 279 downregulated in resistant bendiocarb. (S1A Fig). 455 transcripts were differentially expressed when comparing control to FANG (C vs. S) with 195 upregulated and 260 downregulated in the control versus FANG samples. A lower number of transcripts (197) are differentially expressed in R vs. C with 155 upregulated in R and 42 downregulated in the resistant versus control samples. A similar approach was taken for Ghana *An*. *funestus* where bendiocarb resistance has also been reported. A total of 927 transcripts were differentially expressed between R vs. S in Ghana with 481 up-regulated and 446 downregulated in resistant versus control samples (S1B Fig). 825 transcripts are differentially expressed when comparing Ghana control to FANG (C vs. S) with 456 upregulated and 369 downregulated. 245 were differentially expressed in R vs. C with 90 upregulated and 155 downregulated in resistant versus control samples.

### Malawi

#### Transcripts commonly upregulated in all comparisons (R vs. S, C vs. S and R vs. C)

Analysis of the list of transcripts commonly upregulated in all three comparisons in Malawi revealed the presence of two transposons with the retrovirus-related Pol polyprotein. Of the two, AFUN019813 exhibited the highest fold-change in the bendiocarb resistant (R) vs Fang (S) comparison (FC 79.9) followed by the PiggyBac transposable element-derived protein 4 (AFUN011978) (FC11.1 in R vs. S). These genes were upregulated in the bendiocarb resistant. UDP-glucoronosyl transferase 3A1 was the only transcript from a detoxification gene (AFUN011266) upregulated in the R and C samples in all three comparisons (R vs. S, C vs. S and R vs. C). The microRNA mir-279 was also over-expressed in the three comparisons with the highest fold-change of 7 in R vs. S (Table 1).

**Table 1:** List of resistance associated transcripts commonly over-expressed between bendiocarb resistant (R), unexposed *An*. *funestus* (C) from Malawi and the FANG susceptible (S) strain at fold-change>2 (or 1.5 for R vs. C) FDR<0.05.

Gene ID	R vs.S	C vs.S	R vs.C	Description	
AFUN019813	79.9	37.4	2.1	Retrovirus-related Pol polyprotein from transposon TNT 1–94	
AFUN011266	3.6	2.2	1.7	UDP-glucuronosyltransferase 3A1	
AFUN017296	7.0	4.4	1.6	microRNA mir-279	
AFUN018674	6.1	3.6	1.7	Serine protease easter	
AFUN011978	11.1	6.1	1.8	PiggyBac transposable element-derived protein 4	
AFUN019220	3.8	3.1		ATP-binding cassette transporter (ABC transporter) family A member	
AFUN019401	2.1	2.9		Cytochrome P450, CYP6M4	
AFUN015785	2.2	2.1		Cytochrome P450, CYP6AA2	
AFUN015801	2.5	2.3		Cytochrome P450, CYP6P2	
AFUN020895	6.2	6.0		Cytochrome P450, CYP6P4a	
AFUN001382	3.0	3.4		Cytochrome P450, CYP9J5	
AFUN001383	4.0	4.1		Cytochrome P450, CYP9J5	
AFUN015792	43.3	53.4		Cytochrome P450, CYP6P9a	
AFUN015889	17.0	16.6		Cytochrome P450, CYP6P9b	
AFUN002978	2.5	2.0		Cytochrome P450, CYP314A1	
AFUN015807	2.6	2.6		Glutathione S-transferase, GSTe1	
AFUN015839	3.6	5.1		Glutathione S-transferase, GSTD3	
AFUN016008	3.2	2.8		Glutathione S-transferase, GSTe6	
AFUN000622	2.2	3.0		solute carrier family 23 (nucleobase transporter)	
AFUN021547	2.2	2.3		Sugar transporter SWEET	
AFUN016449	3.1	3.8		Trypsin	
AFUN020614	2.5	2.9		Trypsin	
AFUN003724	2.5	3.1		CCAAT/enhancer-binding protein gamma	
AFUN019110	2.7	3.2		Transcription factor Adf-1	
AFUN008611	6.7	7.1		Transposon TX1 uncharacterized 149 kDa protein	
AFUN020948	2.9		1.9	Chymotrypsin-1	
AFUN015887	3.1		1.7	Gustatory receptor 68a	
AFUN009199	2.6		1.5	Chitin synthase 6	
AFUN007080	2.7		2.2	Caspase-1	
AFUN021252	2.1			Zinc finger protein 91	
AFUN008941	2.5			ATP-binding cassette transporter (ABC transporter) family C member	
AFUN015776	2.1			Cytochrome P450 CYP12F1	
AFUN007186	2.1			Facilitated trehalose transporter Tret1	
AFUN006135	2.1			Cytochrome P450, CYP4C36	
AFUN003300	2.2			Zinc finger protein 420	
AFUN018998	2.0			Zinc finger protein 337	
AFUN000799	2.2			Zinc finger CCHC-type and RNA-binding motif-containing protein 1	
AFUN010918		2.2		Cytochrome P450, CYP6N1	
AFUN014520		2.0		CCR4-NOT transcription complex subunit 7	
AFUN021519		2.1		Zinc finger protein 84	
AFUN021263		2.0		Transcription initiation factor TFIID subunit 8	
AFUN016502		2.2		Sodium/potassium/calcium exchanger 5	
AFUN020670		2.7		Xanthine dehydrogenase	
AFUN015907		2.2		Cytochrome P450, CYP305A3	
AFUN008943			1.7	ATP-binding cassette transporter (ABC transporter) family C member	
AFUN021444			2.0	Zinc carboxypeptidase A 1	

#### Transcripts commonly expressed in R vs. S and C vs. S

The list of transcripts commonly expressed in both R vs. S and C vs. S from Malawi exhibited several cytochrome P450s among which the duplicated *CYP6P9a* (FC 43.3 and 53.4) and *CYP6P9b* (FC 17 and 16.6) were by far the most up-regulated in R versus S samples. Other P450s include three genes located on the same P450 cluster as *CYP6P9a/b* namely, *CYP6P4a*, *CYP6P2*, and *CYP6AA2*. The two transcripts of *CYP9J5* and *CYP6M4* are also commonly upregulated in R and S samples. This list of commonly expressed transcripts between R and C also includes three glutathione s-transferases (*GSTe1*, *GSTe6*, and *GSTD3*), an ATP-binding cassette transporter (AFUN019220), a transposon (AFUN008611), as well as the other gene families such as transporters (AFUN000622, AFUN021547), digestive enzymes (Trypsin), transcription factors (AFUN008611) (Table 1).

#### Transcripts upregulated in other comparisons

The transcripts upregulated in other comparisons (R vs. C and R vs. S; R vs. C and C vs. S etc) in general present low fold-changes less than 3 and include other cytochrome P450s (*CYP6N1*, *CYP4C36*, *CYP12F1*), ABC transporters, digestive enzymes (chymotrypsin 1) as well as transcription factors (Table 1).

#### Co-expression between bendiocarb and permethrin resistance

To detect the genes possibly associated with cross-resistance between carbamates and pyrethroids, the set of transcripts commonly upregulated in both permethrin resistant vs FANG, bendiocarb resistant vs FANG, and also the unexposed vs FANG was identified. This list was again dominated by the duplicated P450 *CYP6P9a* and *CYP6P9b* with the highest FC for the three comparisons (R-Perm vs FANG, R-Ben vs FANG and control vs FANG) in R-Perm, FANG and control (Table A in S2 Table). However, although the retrovirus-related Pol polyprotein AFUN019813 was also commonly upregulated for both insecticide classes (R-Perm vs FANG and R-Ben vs FANG), the fold change was much lower in permethrin resistance (FC11.4) compared to bendiocarb (FC79.9). Other genes are similar to those already predominantly commonly upregulated in R vs. S and C vs. S for bendiocarb.

A direct comparison of permethrin resistant to bendiocarb resistant *An*. *Funestus* revealed no detoxification gene was significantly upregulated in bendiocarb resistant samples compared to permethrin. The retrovirus-related Pol polyprotein AFUN019813 was seven time over-expressed in bendiocarb resistant than in permethrin (Table B in S2 Table). Among the list of transcripts over-expressed in permethrin than bendiocarb resistant *An*. *funestus*, three odorant-binding proteins exhibited the highest fold-change (3.4–5.4) (Table B in S2 Table). Other genes recorded have low fold-change ~2 including two P450s, *CYP6M4* and *CYP6N1*, the arginine-succinate, and the alpha-crystallin B chain (AFUN019875) and a sulfotransferase (AFUN008239).

#### Comparison of field bendiocarb resistant *An*. *funestus* to field fully susceptible *An*. *funestus*

The 12 replicates of *An*. *funestus* from Malawi (3 permethrin alive, 3 bendiocarb alive, 3 DDT alive, 3 unexposed) were pooled with the 4 replicates from the pyrethroid/carbamate resistant FUMOZ and compared them to the bendiocarb susceptible samples from Uganda (3 permethrin alive, 3 DDT alive, 3 unexposed) plus the 4 replicates from FANG fully susceptible laboratory strain. This analysis (Malawi and FUMOZ versus Uganda and FANG) revealed that the duplicated cytochrome P450 *CYP6P9a* (FC 30.4) and *CYP6P9b* (FC 5.2) were the most highly upregulated in the bendiocarb resistant samples (Malawi and FUMOZ) (Table C in S2 Table). Other transcripts included some P450s and the retrovirus-related polyprotein AFUN019813. In contrast, the cytochrome P450 *CYP6P5* was significantly upregulated in the Ugandan populations and this is likely an association with the specific role of this gene in pyrethroid resistance in Uganda.

### Bendiocarb resistance profiling in West Africa (Ghana)

Since a strong contrast has previously been highlighted in pyrethroid resistance between West and Southern Africa, bendiocarb resistance in West Africa (Ghana) where both pyrethroid and carbamate resistance have been detected [13] was also analyzed. No detoxification gene was commonly up-regulated in all three comparisons of R vs. S, C vs. S and R vs. C, with only two chymotrypsin transcripts observed as over-expressed (Table 2). In contrast, the list of transcripts commonly up-regulated in R vs. S and C vs. S revealed several detoxification genes among which the most over-expressed compared to the susceptible samples were the duplicated cytochrome P450 *CYP6P4a* (FC 38.8 and 41.1 in R and C samples respectively) and *CYP6P4b* (FC 16.9 and 17.2 in R and C respectively). Several other P450s genes are overexpressed in R and C compared to S samples although their fold change are lower than that of *CYP6P4a/b*. Among the P450 genes that are overexpressed in R and C versus S samples, *CYP6P5* had the highest fold-change (FC7.0 and 7.5 in R and C samples respectively) followed by *CYP6P9a* (FC 4.7 and 3.6 in the R and C samples respectively) and *CYP6P9b* (FC 6.8 and 6.2 in the R and C samples respectively) although with lower FC than in Southern Africa. Contrary to *An*. *funestus* from Malawi, several GSTs genes are upregulated commonly in R vs. S and C vs. S samples including six epsilon genes from which the *GSTe2* was the most over-expressed (FC9.0 and 9.3 in R and C samples respectively). Other upregulated detoxification genes in the R vs. S and C vs. S samples include a carboxylesterase (AFUN016367), an UDP-glycosyl transferase (AFUN011266). This list also noticeably includes several gustatory receptor proteins (seven), odorant receptors (four) and cuticular proteins (Table 2). The microRNA mir-279 upregulated in *An*. *funestus* from Malawi was also commonly over-expressed in R vs. S and C vs. S *An*. *funestus* from Ghana. Transcripts only up-regulated in other comparisons overall belong to the same gene families as above. When a direct comparison of the bendiocarb resistant was made against permethrin resistant, the list of transcripts over-expressed in the bendiocarb resistant was predominantly made of cuticular proteins, gustatory receptors, odorant binding proteins, odorant receptors. In contrast, the list of transcripts upregulated in permethrin resistant included 2 alpha-crystallin B chain similar to the case in Malawi, two carboxylesterases including AFUN002514 associated with pyrethroid resistance in Cameroon [23]. Several heat shock proteins were also included (Table D in S2 Table).

**Table 2:** List of resistance associated transcripts commonly over-expressed between bendiocarb resistant (R), unexposed *An*. *funestus* (C) from Ghana and the FANG susceptible (S) strain at fold-change>2 (or 1.5 for R vs. C) FDR<0.05.

Gene ID	R_B vs. S	C vs. S	R_B vs. C	Description
AFUN009946	5.2	2.3	2.3	Chymotrypsin-elastase inhibitor ixodidin
AFUN022157	3.6	2.2	1.6	Chymotrypsin-1
AFUN019220	4.8	3.5		ABC transporter family A member
AFUN016367	2.1	2.3		Carboxylesterase
AFUN022110	4.2	5.1		cuticular protein RR-1 family
AFUN009933	2.2	3.2		cuticular protein RR-1 family
AFUN004126	2.8	2.6		cuticular protein RR-2 family
AFUN004316	4.9	4.7		Cytochrome P450, CYP4H17
AFUN006135	2.5	2.4		Cytochrome P450, CYP4C36
AFUN007549	3.2	2.9		Cytochrome P450, CYP9K1
AFUN015830	2.7	2.6		Cytochrome P450, CYP325C
AFUN020895	38.8	41.1		Cytochrome P450, CYP6P4a
AFUN019365	16.9	17.2		Cytochrome P450, CYP6P4b
AFUN001383	3.5	3.2		Cytochrome P450, CYP9J5
AFUN015792	4.7	3.6		Cytochrome P450, CYP6P9a
AFUN015889	6.8	6.2		Cytochrome P450, CYP6P9b
AFUN015888	7.0	7.5		Cytochrome P450, CYP6P5
AFUN002978	2.6	2.3		Cytochrome P450, CYP314A1
AFUN015894	3.9	4.8		Cytochrome P450, CYP4H26
AFUN006858	2.1	2.2		Cytochrome P450, CYP306A1
AFUN015795	2.3	2.3		Cytochrome P450, CYP6M3
AFUN010918	2.6	3.2		Cytochrome P450, CYP6N1
AFUN005715	2.2	2.1		Cytochrome P450, CYP315A1
AFUN016456	2.1	2.3		D7 short form salivary protein
AFUN011266	2.3	2.8		glucosyl/glucuronosyl transferases
AFUN015767	2.4	2.0		Glutathione S-transferase, GSTD1
AFUN015807	3.1	3.9		Glutathione S-transferase, GSTE1
AFUN015808	3.1	3.0		Glutathione S-transferase, GSTE3
AFUN015810	3.6	3.5		Glutathione S-transferase, GSTE4
AFUN015811	2.6	2.9		Glutathione S-transferase, GSTE5
AFUN015839	4.3	4.1		Glutathione S-transferase, GSTD3
AFUN015840	4.3	3.7		Glutathione S-transferase, GSTD10
AFUN016008	3.4	3.4		Glutathione S-transferase, GSTE6
AFUN015809	9.0	9.3		Glutathione S-transferase, GSTE2
AFUN019604	2.0	2.2		Glutaredoxin-3
AFUN015985	5.6	5.7		Gustatory receptor, GR31
AFUN015983	2.9	2.4		Gustatory receptor, GR30
AFUN015855	4.7	4.2		Gustatory receptor, GR60
AFUN015817	3.7	3.1		Gustatory receptor, GR15
AFUN015818	3.7	5.4		Gustatory receptor, GR17
AFUN008425	2.7	2.5		Gustatory receptor, GR18
AFUN003075	2.4	3.2		Gustatory receptor, GR5
AFUN021404	4.2	3.5		Heme peroxidase
AFUN017296	2.9	4.1		microRNA mir-279
AFUN015994	2.0	2.2		Odorant receptor, OR342
AFUN001688	2.9	2.6		Odorant receptor, OR75
AFUN015989	3.8	4.3		Odorant receptor, OR41
AFUN018655	6.2	5.9		Odorant receptor
AFUN022112	3.9		3.5	cuticular protein RR-1 family
AFUN017237	2.7		1.7	microRNA mir-276
AFUN008090	3.6		2.0	odorant receptor, OR36
AFUN007247	2.3		1.9	odorant-binding protein
AFUN016264	2.1			Carboxyesterase
AFUN001382	2.6			Cytochrome P450, CYP9J5
AFUN016010	2.0			Glutathione S-transferase, GSTD1
AFUN004166	2.0			Gustatory receptor, GR48
AFUN015844	2.2			Gustatory receptor, GR51
AFUN009939	2.1			Cuticle Protein 19
AFUN001774		2.4		Glutathione s-transferase, GSTE7
AFUN017071		2.9		microRNA mir-286
AFUN011830		2.4		odorant receptor, OR22
AFUN000622		2.3		Solute carrier family 23

#### Gene Ontology enrichment

Bendiocarb resistant *An*. *funestus* from Malawi/Southern Africa showed significant enrichment of gene ontologies associated with cytochrome P450 genes (heme binding and monooxygenase activities) in genes over-expressed relative to the fully susceptible FANG (Malawi/FUMOZ versus FANG). Similarly when compared against *An*. *funestus* from Uganda (Malawi/FUMOZ versus Uganda), gene ontology revealed that the cytochrome P450 genes (heme binding and monooxygenase activities) were significantly enriched. These GO terms included heme binding, tetrapyrrole binding, oxidoreductase activity and iron ion binding (S2 Fig).

### qRT-PCR

**Fig 1:**
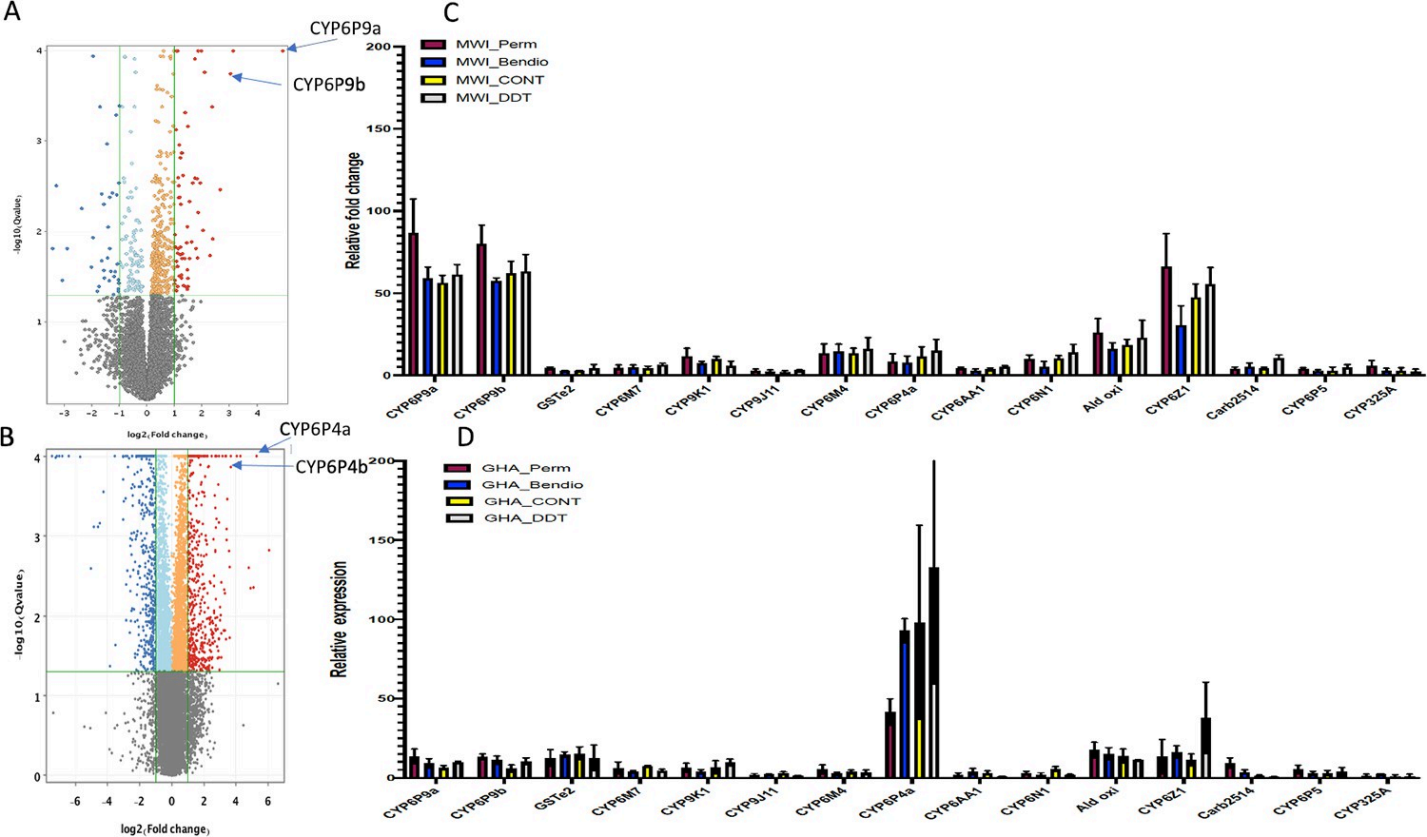
Transcription profiling of bendiocarb resistance. A) Volcano plot of differential gene expression between bendiocarb-exposed samples from Malawi and FANG showing the high over-expression from *CYP6P9a* and *-b*. B) Volcano plot of differential gene expression between bendiocarb-exposed samples from Ghana and FANG showing the high over-expression from *CYP6P4a* and -*b*. C) qRT-PCR validation of the expression profile of the main detoxification genes differentially expressed between resistant and susceptible bendiocarb samples with RNAseq in Malawi; MWI; Malawi, Perm is permethrin, Bendio is bendiocarb. D) is for Ghana.

Quantitative real-time PCR (qRT-PCR) was performed to confirm differential expression observed between bendiocarb resistant samples and other resistance phenotypes and unexposed control samples in Malawi (Fig 1C) and Ghana (Fig 1D). This was done for fifteen detoxification genes that exhibited the highest expression from RNAseq including twelve P450 genes, one glutathione S-transferase gene (*GSTe2*), a carboxlesterase gene (AFUN002514) and one aldehyde oxidase gene (AFUN004380). Overall, the qRT-PCR results correlated strongly with the RNAseq data (R^2^ = 0.776; P<0.001) (S3C Fig) supporting the over-expression patterns observed for bendiocarb, permethrin and DDT resistant and control samples when compared to FANG *An*. *funestus*. In Malawi, the duplicated P450s *CYP6P9a* and *-b* were the most overexpressed genes (R vs. S *An*. *funestus* samples) with a slightly greater FC in permethrin resistant (FC 86.5±20.7 and FC 79.8±11.5 for *CYP6P9a* and -*b* respectively) than in bendiocarb resistant, DDT–resistant and unexposed control versus susceptible *An*. *funestus* (Fig 1C). The *CYP6Z1* gene previously shown to metabolise bendiocarb was overexpressed in resistance versus susceptible samples with the FC being greater in permethrin resistant (66.1±20.2 in R-Perm vs FANG) than bendiocarb resistant (30.4±11.8 in R-Bendiocarb versus FANG). For *An*. *funestus* from Ghana, the *CYP6P4a* gene was by far the most over-expressed in the resistant and control samples as observed in RNAseq data (FC40-130) with the variation explained by high SD (Fig 1D).

### Detection of variants associated with cross-resistance between carbamate and pyrethroid using targeted sequencing

To detect the polymorphisms associated with bendiocarb resistance in *An*. *funestus*, a targeted sequencing approach was also used to enrich a genomic regions that is likely associated with resistance to insecticides. A total of 3,059,528bp of the 1302 sequence capture regions was successfully sequenced in 68 individual *An*. *funestus*. The coverage was high and ranged between 222 and 359 fold.

**Fig 2:**
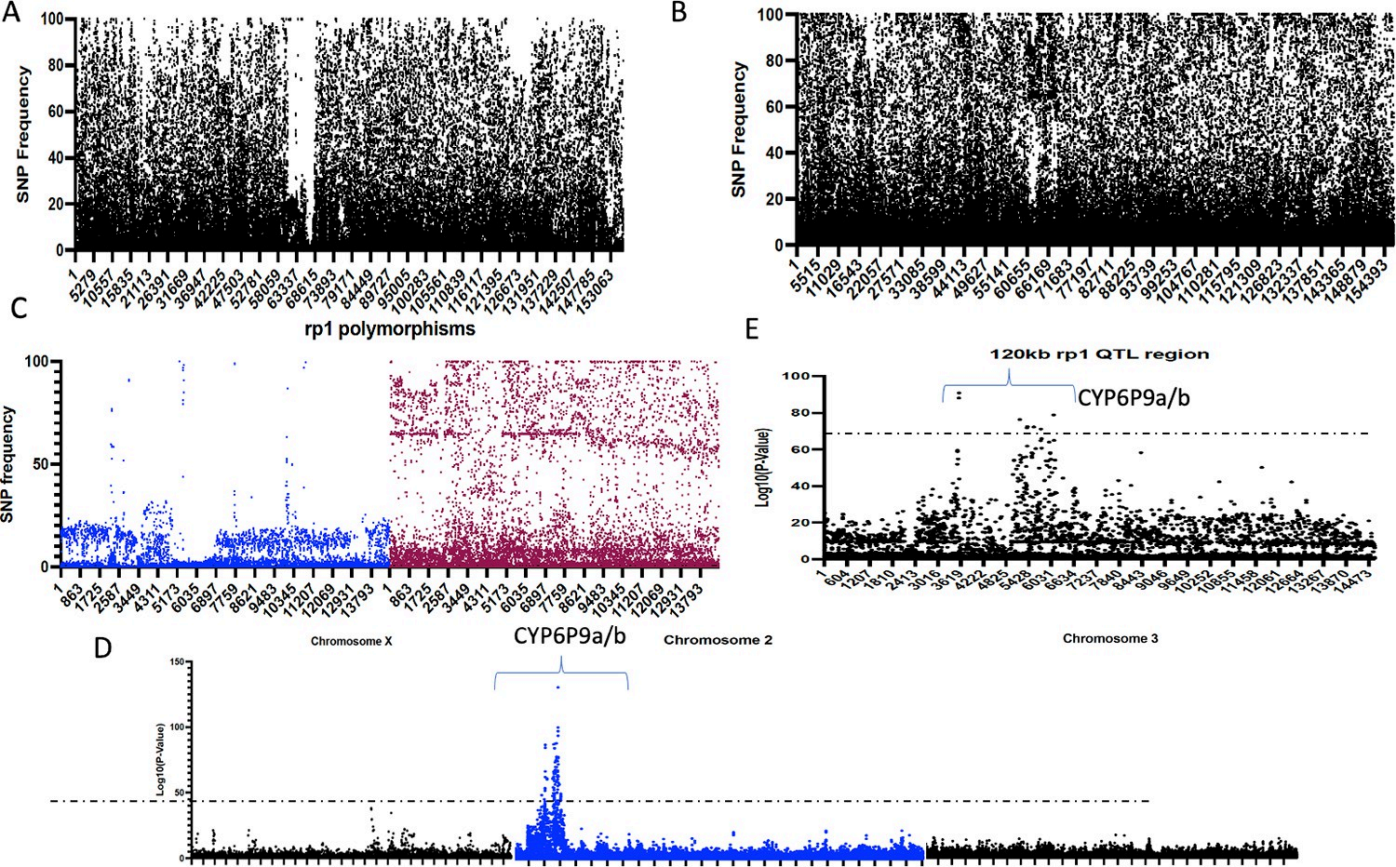
Contrasting genetic diversity between carbamate resistant and susceptible *An*. *Funestus* identifies *CYP6P9a/b* as tightly associated genes to cross-resistance. A) Reduced diversity in Southern African samples is detected in 2R chromosome but not in Uganda. B) close-up analysis of the 120kb sequence spanning the known pyrethroid resistance genomic loci reveals extensive reduced diversity in Southern African populations compared to Ugandan bendiocarb susceptible. C) Comparative allele frequency of variants spanning the rp1 QTL region. The blue represents Southern African samples and the purple is for the Ugandan *An*. *Funestus*. D) The Manhattan plot representing the genome-wide association study of bendiocarb resistance comparing Southern African bendiocarb resistant vs Uganda fully susceptible *An*. *Funestus*. The–log10p-values are plotted against the position on each chromosome (unpaired t-test). The horizontal dashed line shows the genome-wide significance cut-off at FDR of 0.05. E) Manhattan plot for the 120kb genomic region spanning the rp1 QTL between the same Southern Africa and Uganda samples.

A total of 156,698 polymorphic sites were detected across all 68 *An*. *funestus* analysed. The Southern African samples exhibited lower polymorphisms compared to the reference genome which is expected as both are from Southern Africa. Detection of the SNPs significantly associated with bendiocarb resistance was performed firstly using the differential SNP frequency analysis implemented in Strand NGS. Comparing the mean allele frequency of the bendiocarb resistant *An*. *funestus* from Malawi/FUMOZ to the susceptible *An*. *funestus* from Uganda/FANG revealed a major region with the highest contrast between both populations as the Southern African *An*. *funestus* exhibited a reduced diversity on chromosome 2 (Fig 2A), whereas this is not the case in Uganda-FANG (Fig 2B). Because a 120kb fragment of the rp1 genomic regions was also enriched and sequenced for these samples (Malawi, FUMOZ, Uganda and FANG), the polymorphism of this entire region between Southern African and Ugandan samples was compared. This revealed an extensive loss of diversity across the 120kb of the rp1 genomic region in Southern African bendiocarb resistant *An*. *funestus* with the highest reduced diversity seen across the 14kb region spanning *CYP6P9a* and *CYP6P9b* (Fig 2C).

Unpaired t-test of allele frequency in both sample sets broadly detected two genomic regions significantly associated with bendiocarb resistance. The hit with the lowest P-value (P = 1.55 x 10^−130^) is located on the 2R chromosome spanning the known rp1 QTL region with several other SNPs also significantly differentially distributed between these populations in the locus (Fig 2D). Many of these SNPs were located on the *CYP6P9a* and *CYP6P9b* genes (Table A in S3 Table). The second locus in on the X chromosome at the vicinity of the *CYP9K1* P450 gene (P = 7.26.10^−39^) (Fig 2D). An unpaired t-test performed for the allele frequencies difference of the recorded polymorphisms across the 120kb rp1 region also revealed that the most significant SNPs are located across the duplicated *CYP6P9a* and *CYP6P9b* genes (Fig 2E).

SNPs significantly associated with bendiocarb resistance were detected using the differential SNP frequency analysis implemented in Strand NGS assuming a cut-off of 1–25% frequency in Southern African *An*. *funestus* and 75%-100% for Uganda-FANG (as these are different from the reference genome from FUMOZ). A total of 3999 SNPs were found significantly distributed between these samples belonging to 148 genes (37 on the X chromosome, 22 on chromosome 3, and 78 on Chromosome 2). Of these significant SNPs, 2084 were detected in the AFUN020405 transcript coding for the solute carrier family 8 (sodium/calcium exchanger) (AGAP002859 in *An*. *gambiae*). This is a large gene of 109614bp and 8368bp of the coding region spread over the entire *rp1* locus overlapping many other genes, thus leading to double counting of the SNPs in this region. When AFUN020405 SNPs are removed, there were 1095 significant SNPs with a large portion still located around the *rp1* QTL region harboring *CYP6P9a/b* on the 2R chromosome. When assessing the non-synonymous SNPs, out of 162 detected, 145 are located on the 2R chromosome notably from the cluster of CYP6 cytochrome P450s that span the *rp1* resistance loci notably *CYP6AA1* (9), *CYP6P2* (9) and *CYP6P9a* (4) and *CYP6P9b* (2) (Table B in S3 Table and S4A and S4B Fig). Interestingly, the acetylcholinesterase gene (*ace*-1) known to be associated with carbamate resistance exhibited a significant allelic frequency profile differences between Southern Africa and Uganda bendiocarb susceptible *An*. *funestus* suggesting a role of target-site resistance to carbamate resistance as shown previously [27] although the N485I mutation was not differentially selected.

### Determination of bendiocarb-metabolising ability of *CYP6P9a* and *CYP6P9b*

#### Pattern of expression of recombinant *CYP6P9a* and *CYP6P9b*

**Fig 3:**
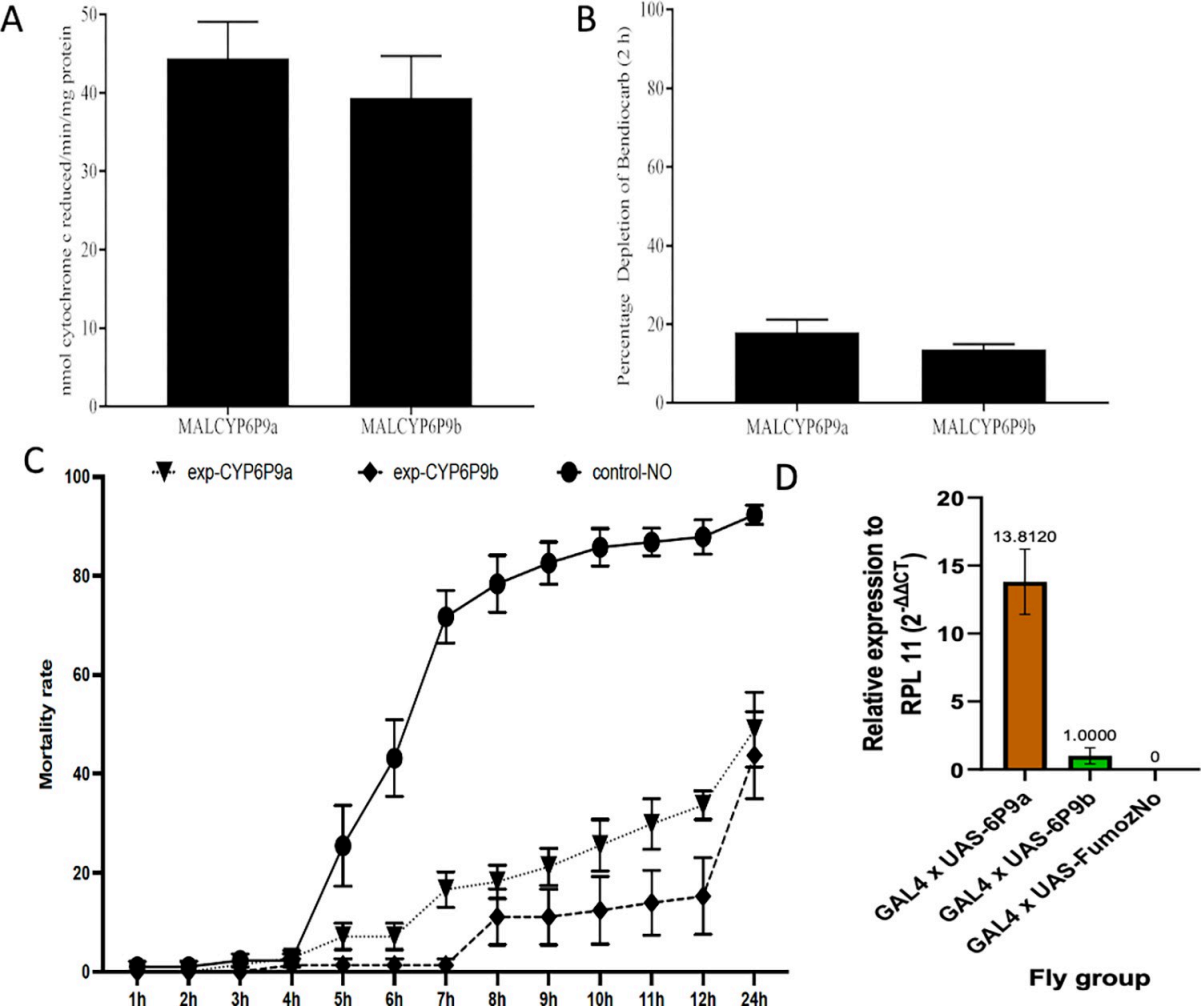
Functional validation of the ability of *CYP6P9a/b* to confer resistance to carbamates. A) Determination of cytochrome P450 reductase activity using a model substrate, *cytochrome c*. All results are average of three replicates ± standard error of the means. B) Metabolic activities of the recombinant *An*. *funestus CYP6P9a* and *CYP6P9b*. Depletion of 20 μM bendiocarb in metabolic assays with recombinant CYP6P9a and–b (SEM = 3). C) Transgenic expression of CYP6P9a/b in *Drosophila melanogaster* using the GAL4/UAS system. The observed mortality pattern of GAL4 x *UAS-CYP6P9a* and GAL4 x *UAS-CYP6P9b* flies exposed to 0.007% bendiocarb. D) The relative expression of the CYP6P9a and b transgenes in transgenic *D*. *melanogaster* Act5C-CYP6P9a/b strains and the control sample with no transgene expression. The data shown are the mean ± SEM (n = 3).

Since RNAseq and target enrichment sequencing both associated the duplicated P450s *CYP6P9a* and *CYP6P9b* with bendiocarb resistance, an *E*. *coli* heterologous expression followed by metabolism assays with insecticides were used to functionally validate their roles. The optimal expression of these recombinant P450s was obtained at 40 h post induction, with P450 spectral activities of 13.8 nmol/ml (P450 content of 0.53 nmol/mg protein) and 13.2 nmol/ml (0.49 nmol/mg protein) for *CYP6P9a* and *-b*, respectively. The P450 contents obtained were similar to the previously expressed membranes [40]. The recombinant *CYP6P9a* and *-b* exhibited P450 reductase activity of 44.39 nmol cytochrome c reduced/min/mg protein and 39.38 nmol cytochrome c reduced/min/mg (Fig 3A), which were lower than established for the same alleles expressed in previous work [40].

#### Recombinant *An. funestus CYP6P9a* and -b metabolism of bendiocarb

Recombinant *CYP6P9a* metabolise bendiocarb with low activity, generating polar metabolites at the beginning of the chromatogram on incubation with NADPH+, consistent with previous observations from the recombinant *A*. *funestus CYP6Z1* [27]. At 2 h of incubation 18.04% ± 3.14 of bendiocarb was depleted by *CYP6P9a* (p = 0.08, NADPH+ vs NADPH- incubations) (Fig 3B). Depletion of 13.65% ± 1.29 was obtained with recombinant *CYP6P9b* as well, after 2 hours of incubation.

### Assessing the role of *CYP6P9a* and *CYP6P9b* in carbamate resistance using transgenic *Drosophila melanogaster*

To establish whether the up-regulation of each of *CYP6P9a and CYP6P9b* genes alone can confer resistance to carbamates, previously generated transgenic *D*. *melanogaster* [using the GAL4/UAS system under the ubiquitous Act5C-GAL4 driver (A5C-CYP6P9a; Act5C-CYP6P9b)] were used [21]. Bioassays performed with 0.007% bendiocarb revealed that flies expressing *CYP6P9a* and *CYP6P9b* were significantly more resistant to bendiocarb than the control flies, with average mortalities at 24 h of 48.93% for *CYP6P9a* (P< 0.001) and 43.73% for *CYP6P9b* (P< 0.001) compared to the control (92.31%) (Fig 3C and Table A in S4 Table). These results indicate that the over-transcription of each of these duplicated genes is alone sufficient to confer resistance to carbamates as they were also shown previously to do the same for pyrethroids [21,39].

The expression of each gene was confirmed by qRT-PCR showing their significantly increased expression in the respective transgenic flies (Fig 3D). Furthermore, the specificity of the primers used was confirmed as the *CYP6P9a* resistant gene exhibited a 13-fold more over-expression in in the GAL4 x UAS-*CYP6P9a Drosophila* fly group, compared to the GAL4 x UAS-*CYP6P9b* fly group (P = 0.0008, Student’s t-test). There was no Ct value for the control group indicating a lack *CYP6P9a* expression.

### Assessing the correlation between *CYP6P9a*/6.5kb insertion and ability to survive exposure to carbamates

#### Susceptibility and synergist tests with F_4_ female hybrid adult *An*. *funestus*

Bioassays carried out using 600 randomly mixed F_4_ females, showed a mortality rate of 85±14.4% recorded for propoxur (0.1%) and 84±9.7% for bendiocarb (0.1%) after 1 hour exposure. A much lower mortality rate of 37±9.7% was observed for deltamethrin-exposed *An*. *funestus* than for carbamate exposed, indicating higher resistance to pyrethroids (S5 Fig and Table B in S4 Table). However, susceptibility was almost fully recovered (from 85±14.4% to 98±3.5%) when the female *An*. *funestus* were pre-exposed to 4% (piperonly butoxide) PBO followed by bendiocarb. This indicated that P450s are playing a role in carbamate resistance in *An*. *funestus*.

#### Correlation between 6.5kb structural variant resistant allele genotypes and carbamate resistance; i) bendiocarb

**Fig 4:**
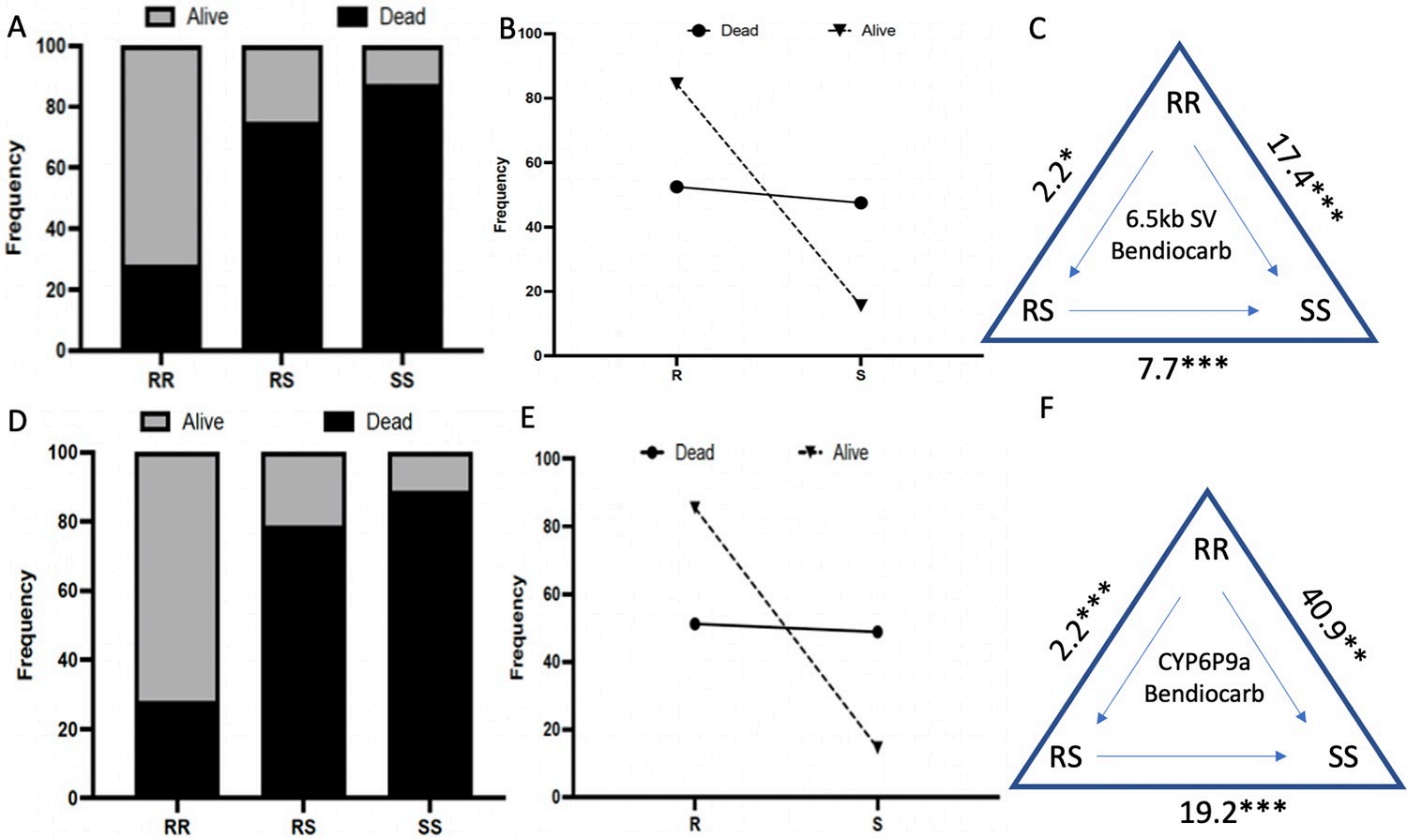
Correlation between genotypes at *CYP6P9a/6*.*5kb SV* and carbamate resistance. (A) Distribution of *CYP6P9b* genotypes between dead and alive *An*. *funestus* after exposure to 0.1% bendiocarb using WHO bioassay showing that CYP6P9a_R significantly allows *An*. *funestus* to survive exposure to this carbamate. (B) Correlation between frequency of CYP6P9b-R and ability to survive exposure to bendiocarb. C) Estimation of odds ratio (OR) and associated significance between different genotypes and the ability to survive exposure to bendiocarb. Ors are given with asterisks indicating level of significance. The arrow within the triangle indicates the direction of OR estimation. For example, Individuals that are RR are 17.4 times more likely to survive exposure to bendiocarb that those carrying two copies of the susceptible allele (SS). D), E) and F) are the same respectively for *CYP6P9a*.

A significant difference in the distribution of genotypes of the 6.5kb SV was observed between dead and alive *An*. *funestus* after exposure to bendiocarb (Chi^2^ = 84.4; P<0.0001) (Fig 4A) with a greater percentage of the homozygote with the 6.5 kb SV (SV+/SV+) genotype recorded in the alive (72%) than in the dead *An*. *funestus* (28%). A strong association was found between the 6.5kb SV and the ability to survive exposure to bendiocarb (Fig 4A and 4B). *An*. *funestus* homozygous for the 6.5kb SV (SV+/SV+) had a greater chance of surviving exposure to bendiocarb (72% survived) than the heterozygous *An*. *funestus* (SV+/SV-) (25% survived) (OR = 7.7, CI = 4.1–14.5, P<0.0001 Fisher’s exact test) and the homozygous without the structural variant (SV-/SV-) (13% survived) (OR = 18, CI = 8.6 to 37.5, P<0.0001 fisher’s exact test) (Fig 4C and Table A in S5 Table). This indicates that *An*. *funestus* with the homozygous form of the structural variant (SV+/SV+) have an increased advantage to survive exposure to bendiocarb. A weaker but still significant association was found between the SV+/SV- and the ability to survive exposure to bendiocarb when compared to the SV-/SV- (OR = 2.3, CI = 1.1 to 4.9, P = 0.031) (Fig 4C). Overall, possessing a single 6.5kb SV allele confers a significant ability to survive exposure to bendiocarb than for the SV- allele (OR = 4.75, CI = 2.4–9.2, P<0.0001 (Table A in S5 Table and Fig 4B).

**ii) Propoxur:** A similar pattern was observed for *An*. *funestus* exposed to proxopur as a significant difference in the distribution of genotypes of the 6.5kb SV was observed between dead and alive *An*. *funestus* after exposure to propoxur (Chi^2^ = 100.9; P<0.0001) (S6A Fig) with 67% of the homozygote SV+/SV+ genotype recorded for the alive *An*. *funestus* while only 33% for the dead. A strong association was found between the 6.5kb SV and the ability to survive exposure propoxur (S6A and S6B Fig). *An*. *funestus* homozygous for the 6.5kb SV (SV+/SV+) had a greater chance of surviving propoxur exposure (67% survived) than the heterozygous (48% survived) (OR = 2.2, CI = 1.23–3.88, P = 0.013 Fisher’s exact test) and the homozygous without the structural variant (SV-/SV-) (none survived) (OR = 198, CI = 26.4–1482.6, P<0.0001 Fisher’s exact test) (S6C Fig). This indicates that *An*. *funestus* with the homozygous form of the structural variant (SV+/SV+) have an increased advantage to survive exposure to propoxur. A greater chance of survival to propoxur was also found in *An*. *funestus* heterozygous (SV+/SV) when compared to those homozygous without the structural variant (SV-/SV-) (OR = 90.3, CI = 12.1–672, P<0.0001 fisher’s exact test) (Table B in S5 Table and S6C Fig). Overall, possessing a single 6.5kb SV allele confers a significant ability to survive exposure to propoxur than for the SV- allele (OR = 2.8, CI = 1.5–5.1, P<0.0001 (Table B in S5 Table and S6B Fig).

**b- Correlation between *CYP6P9a* genotypes and carbamate resistance**

#### S) Bendiocarb

A significant difference in the distribution of genotypes of the *CYP6P9a* was observed between dead and alive *An*. *funestus* after exposure to bendiocarb (Chi^2^ = 94.5; P<0.0001, Chi-square) (Fig 4D and 4E) with 72% of the *CYP6P9a* homozygote resistant (RR) genotype recorded in the alive group vs 28% in the dead group. Contrarily, the heterozygous genotype (RS) for *CYP6P9a* was greater in the dead (79%) than in the alive (21%). A majority of the SS genotype was found in the dead group (89%) than in the alive group (11%). A stronger association was found between the *CYP6P9a* homozygous resistant (RR) and the ability to survive exposure to bendiocarb compared to the heterozygous resistant (RS) (OR = 9.7, CI = 5.05–18.5, *P*< 0.0001 Fisher’s exact test) and homozygous susceptible (SS) (OR = 20.8, CI = 9.6–44.6, *P*< 0.0001) (Fig 4F). A significant association between the RS genotype to bendiocarb survival was also observed when compared to the SS genotype (OR = 2.15, CI = 0.97–4.7, *P*<0.04 Fisher’s exact test) (Table A in S5 Table). Thus, *An*. *Funestus* homozygous resistant (RR) for the *CYP6P9a* resistant allele have a much greater chance of surviving exposure to bendiocarb than those with the heterozygous resistant (RS) and homozygous susceptible genotype (SS). Overall, possessing a single CYP6P9a_R allele confers a significant ability to survive exposure to bendiocarb than for the susceptible CYP6P9a_S (OR = 5.5, CI = 2.8–10.8, P<0.0001 (Table A in S5 Table and Fig 4E).

#### ii) Propoxur

A significant difference in the distribution of genotypes of the *CYP6P9a* was also observed between dead and alive *An*. *funestus* after exposure to propoxur (Chi^2^ = 127.9; P<0.0001, Chi-square) (S6D and S6E Fig) with the proportion of *CYP6P9a* homozygote resistant (RR) greater in the alive (78%) than in the dead (22%). However, the heterozygote genotype (RS) occurred more frequently in dead (61%) than alive (39%) *An*. *funestus*. The homozygote susceptible (SS) genotypes for the *CYP6P9a* were all found in the dead. A stronger association was found between the *CYP6P9a* homozygous resistant and the ability to survive exposure to propoxur compared to the heterozygous resistant (RS) (OR = 63.3, CI = 8.5–472.6, P<0.0001 Fisher’s exact test) and homozygous susceptible (SS) (OR = 351, CI = 46.3–2661.6, P<0.0001) (Table B in S5 Table and S6F Fig). This thus indicates that *An*. *funestus* with the homozygous genotype for the *CYP6P9a* have a much greater chance of surviving exposure to propoxur than those with the homozygous susceptible genotype (SS). Overall, possessing a single CYP6P9a_R allele confers a significant ability to survive exposure to propoxur than for the susceptible CYP6P9a_S (OR = 6, CI = 3.2–11.3, P<0.0001 (Table B in S5 Table and S6E Fig).

### Combined effect of the 6.5kb structural variant and *CYP6P9a* on carbamate resistance

**Fig 5:**
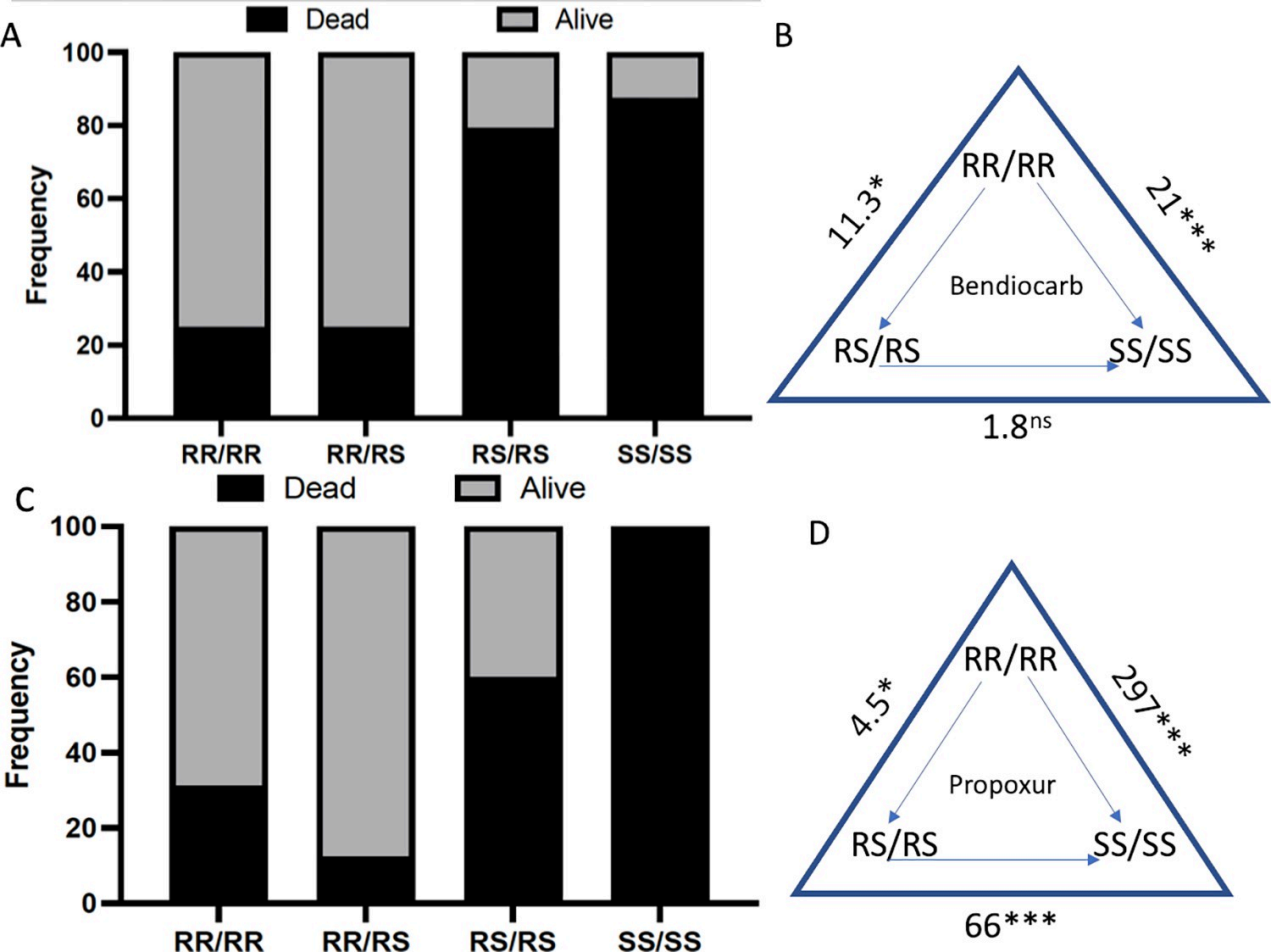
Combined effect of the 6.5kb insertion and *CYP6P9a* on carbamate resistance. A) Distribution of combined genotypes between bendiocarb resistant and susceptible *An*. *funestus* showing that genotypes at both loci combined to additively increase the ability to survive after exposure to bendiocarb. B) Double homozygote resistant *An*. *Funestus* are significantly more likely to survive exposure to bendiocarb than double homozygote susceptible (SS) (Odds ratio 21; P<0.001) and than heterozygotes (Odds ratio 11.3; P<0.01). C and D) are for propoxur respectively.

The combined effect of the 6.5kb SV and *CYP6P9a* on the ability to survive exposure to carbamates was further assessed. An increased survivorship was observed when the 6.5kb SV was combined with the *CYP6P9a* for both carbamates (Fig 5A). Results showed that *An*. *funestus* double homozygous resistant for both alleles (SV+/SV+/RR) had a greater chance to survive exposure to bendiocarb when compared to the *An*. *funestus* double homozygous susceptible for both alleles (SV-/SV-/SS) (OR = 21, CI = 2.459 to 244.6, P = 0.0026) and *An*. *funestus* double heterozygous resistant for both alleles (SV+/SV-/RS) (OR = 11.5, CI = 3.361 to 34.84, P<0.0001 Fisher’s exact test) (Fig 5B). However, there was no significant difference between *An*. *funestus* double homozygous resistant and *An*. *funestus* homozygous resistant for one gene and heterozygous resistant for the other gene (Table A in S5 Table). This indicates that there is an added survival advantage in *An*. *Funestus* possessing the two resistant genotypes (Fig 5B).

A similar pattern was also observed in *An*. *Funestus* exposed to propoxur. Genotype/phenotypes analyses revealed an added advantage to *An*. *funestus* double homozygous resistant for both alleles (SV+/SV+/RR) as they had a greater chance to survive exposure to propoxur when compared to the *An*. *funestus* double homozygous susceptible for both alleles (SV-/SV-/SS) (OR = Infinity, CI = 3.411 to Infinity, P = 0.0007) (Fig 5C and 5D). However, there was no significant difference between the *An*. *funestus* double homozygous resistant for both genes and *An*. *funestus* double heterozygous resistant for both alleles (SV+/SV-/RS) (OR = 3.215, CI = 0.9084 to 10.76, P = 0.0717 Fisher’s exact test) (Table B in S5 Table).

## Discussion

Elucidating the molecular basis of resistance to insecticides and deciphering patterns of cross-resistance between different insecticide classes is key to implementing suitable resistance management strategies to improve vector control. This study has characterized the genetic basis of carbamate resistance in the major malaria vector *An*. *Funestus* establishing a strong pattern of cross-resistance with pyrethroids primarily driven by the duplicated cytochrome P450 *CYP6P9a* and *CYP6P9b*.

### 1-RNASeq-based transcription analysis supports a major role of P450s in carbamate resistance in *An*. *Funestus*

RNAseq analysis revealed a predominance of cytochrome P450s among the detoxification genes which are overexpressed in relation to bendiocarb resistance in *An*. *funestus* from both Malawi and Ghana similar to the previous observation in *An*. *Gambiae* [14,44]. The P450s belonging to the CYP6P cluster were among the most over-expressed genes associated with carbamate resistance in *An*. *funestus* as was also reported for pyrethroid resistance in *An*. *Funestus* [21–23] and *An*. *Gambiae* [14,44,45]. This predominance of P450s in these populations correlates with piperonyl butoxide (PBO) synergist assays that were performed in the field and revealed that there was a significant recovery of susceptibility to carbamates after exposure to PBO in *An*. *Funestus* populations from Malawi [7] and Mozambique [8,46]. Furthermore, a field study in five locations in Zambia revealed that populations of *An*. *Funestus* concomitantly exhibited a recovery of susceptibility to both pyrethroids and carbamates after PBO exposure [47] suggesting that P450 genes play a crucial role in resistance to both insecticide classes. But no transcriptomic work was carried out to identify the overexpressed P450 genes. The question, therefore, is whether the P450s involved in carbamate and pyrethroid resistance are the same. Among P450s, the duplicated *CYP6P9a*/*b* and *CYP6P4a/b* genes were predominant in *An*. *funestus* from Southern Africa and West Africa respectively. This marked geographical difference has previously been reported in *An*. *Funestus* in relation to pyrethroid resistance, which is likely to be associated with restriction to gene flow or lack of dispersal time since emergence and differences in local selection processes [23,48].

The high over-expression of *CYP6P9a* and *-b* genes in bendiocarb resistant in *An*. *funestus* from Malawi through RNAseq is similar to what has previously been reported with microarray [27] supporting their role in carbamate resistance. Alternatively, there is a confounding effect of the underlining pyrethroid resistance also seen in these populations. This is the challenge of deciphering the molecular basis of resistance to an insecticide class when dealing with multiple resistance. To disentangle the contribution of candidate genes to one or many resistance can be challenging in situations of such multiple resistance as seen in both Malawi and Ghana where mosquito populations are resistant to pyrethroids, carbamates, and DDT [7,13]. However, the fact that *CYP6P9a* and *CYP6P9b* are by far the most overexpressed genes in both carbamate and pyrethroids resistance in Malawi is a strong indication of their role in cross-resistance. This was observed for P450 genes such as *CYP6P3* and *CYP6M2* in *An*. *Gambiae*, which were both over-expressed in multiple resistant *An*. *Gambiae* populations with high levels of both pyrethroid and carbamate resistance in Ivory Coast [14]. Over-expression of *CYP6P4a* and *-b* genes in *An*. *funestus* from Ghana is a further indication of the resistance shift between African regions as seen previously for pyrethroids indicating that the underlying molecular basis of bendiocarb resistance also varies geographically. The role of *CYP6P4a* and *-b* genes remains to be validated in carbamate resistance. However, orthologues of these genes are also ove–expressed in pyrethroid resistant *An*. *Arabiensis* [49], in *An*. *Gambiae* [44] and in *Aedes albopictus* resistant to pyrethroid and bendiocarb (*CYP6P12*) [50].

### 2-Targeted deep sequencing reveals a major contribution of the *rp1* pyrethroid resistance loci in carbamate resistance

Use of targeted deep sequencing with Sureselect approach revealed a strong association of the genomic region spanning the *rp1* locus which has previously been shown to drive pyrethroid resistance in Southern African populations of *An*. *Funestus* [23,51]. Several lines of evidence highlighted the strength of the association between *rp1* and carbamate resistance including; i) a major signal was located in 2R chromosome in a region spanning *rp1* when comparing *An*. *Funestus* resistant and susceptible to bendiocarb; ii) majority of polymorphisms associated with bendiocarb resistance including nonsynonymous SNPs are found in the cluster of CYP6 P450s on the *rp1* region; iii) when comparing all Southern Africa vs Uganda (where there is no carbamate resistance), *rp1* was still the main genetic loci associated. The consistency of the association of the *rp1* and *CYP6P9a/b* with carbamate resistance correlated well with RNAseq differential expression results. This highlights the usefulness of targeted sequencing in detecting resistance loci as recently done for the detection of *CYP9K1* as a major driver of pyrethroid resistance in Ugandan *An*. *funestus* populations [52]. Similarly, target enrichment sequencing notably with the SureSelect approach was successfully used in *Aedes aegypti* to detect loci associated with pyrethroid resistance [53]. Evidence of reduced diversity around *rp1* in bendiocarb resistant *An*. *Funestus* was also striking and similar to the extensive reduced diversity observed for the pyrethroid resistant *An*. *Funestus* in Southern Africa [48,54] further supporting the power of genomic analyses in detecting resistance loci. Although, this reduced diversity is now fixed across all *An*. *Funestus* Southern African populations. Such low diversity was also the case in *An*. *Gambiae* with the Ag1000G that detected signatures of selective sweep associated with major resistance loci [55]. In *An*. *Funestus* the association of such reduced diversity with resistance phenotypes was further shown by the striking difference observed from whole-genome sequencing between *An*. *Funestus* collected in 2002–2004 in Mozambique and Malawi before scale-up of LLINs and those in 2016 post-LLINs. This was shown by the fact that the genomic region spanning the 2R Chromosome which harbours the rp1 exhibited an extensively reduced diversity post- in contrast to pre-LLINs [48,54].

### 3-*CYP6P9a* and *CYP6P9b* are functionally capable of metabolizing carbamates

Although genetic associations are important, it is essential to functionally establish the ability of specific genes to confer resistance to an insecticide. Here, it was shown that both recombinant *CYP6P9a* and *-b* can confer resistance to bendiocarb and propoxur in addition to their pyrethroid metabolising ability previously demonstrated [21,39,40] providing a robust evidence that both genes are conferring a cross resistance between carbamates and pyrethroids. Several *An*. *funestus* P450s including *CYP6Z1*, *CYP6AA1*, and *CYP9J11* have been functionally validated as cross resistance genes and associated with multiple resistance in this major malaria vector. For example, a comparable depletion of bendiocarb (23.42% ± 4.02, P = 0.08) has been established with recombinant *An*. *funestus CYP6AA1* [56], while higher and significant depletions were obtained with *CYP9J11* (38.34% ± 7.01, P < 0.05) [26] and *CYP6Z1* (54.72% ± 0.45, P < 0.05) [27]. Similar to the observations with the above P450s, for *CYP6P9a* and *-b*, similar polar metabolites were observed eluting at the beginning of the chromatogram. These findings suggest that the P450s from the CYP6 sub-family are responsible for bendiocarb resistance in the field populations of *An*. *funestus* in Southern Africa, each contributing its low activity, which taken together result in detoxification of this carbamate insecticide. However, the bendiocarb depletion rates obtained for *CYP6P9a* and *-b* were lower than for pyrethroids depletion, suggesting that the metabolism of carbamates by these enzymes could be slower than for pyrethroids. In support of this was the higher mortality to carbamates observed in field populations compared to pyrethroids. For example the 2014 susceptibility survey of the Chikwawa populations of *An*. *funestus* indicated a mortality of 13±5.3% for permethrin and 1.8±1.8% for deltamethrin but a higher rate for bendiocarb at 30.1±5.1% [7]. A similar trend was observed in 2017 in Mozambique [8]. It cannot also be ruled out that *CYP6P9a/b* could be conferring resistance by other mechanisms such as sequestration alongside metabolic action, as is the case for *CYP6Z2* in *An*. *gambiae* [57]. The lower metabolism of carbamates by *CYP6P9a/b* compared to pyrethroids is similar to the case of *CYP6M2* in *An*. *gambiae* which has been shown to confer cross resistance to carbamates and pyrethroids but its *in vitro* metabolism of carbamates was not significant. These lower depletion rates with carbamates with recombinant P450 enzymes could also suggest the need to further optimise the carbamate metabolism assays. In the case of *An*. *funestus*, future efforts should try to use the *An*. *funestus* cytochrome P450 reductase (CPR) to improve such metabolism. This is important as studies have shown that co-expression of a P450, with its homologous redox partner can be a requirement for metabolism and resistance, for example, *CYP392A16* and its CPR were associated with abamectin resistance in a two-spotted spider mite [58].

The ability of *CYP6P9a/b* to confer carbamate resistance was even stronger with GAL4/UAS transgenic expression in *Drosophila* flies demonstrating that over-transcription of both genes alone is sufficient to confer carbamate resistance. Similar results have been observed for other *An*. *funestus* genes such as *CYP6Z1* [27] and *CYP9J5* (previously called *CYP9J11* in *An*. *funestus)* [26] for which transgenic flies expressing them also exhibited a low mortality compared to control. The major difference is that contrary to *CYP6Z1* and *CYP9J5*, both *CYP6P9a/b* are also highly over-expressed in the field populations highlighting the major role they play in observed resistance. Other genes such as *CYP6P3* and *CYP6M2* have also been shown to confer bendiocarb resistance via transgenic expression in *Drosophila* in addition to pyrethroid resistance for both genes, and DDT resistance for *CYP6M2* expression [14]. However, the greater implication of *CYP6P9a/b* in pyrethroid resistance was also evident with transgenic expression, as these flies could withstand a dose of 2% (wt/vol) permethrin and 0.15% (wt/vol) deltamethrin [21] while for bendiocarb the dose was far lower (0.007% (wt/vol). Nevertheless, the role of *CYP6P9a/b* in carbamate resistance is further supported by the fact that high resistance to carbamates in *An*. *funestus* (30% to 50% mortality) is found mostly in Southern Africa where *CYP6P9a* and *CYP6P9b* are by far the most overexpressed in resistant versus susceptible populations [8] while in other parts of Africa such as in central (Cameroon) and Eastern (Uganda) Africa, resistance to carbamates still remains at a low level with mortality levels between 85–98% [3,9,18,19]. Equally, the FUMOZ resistant laboratory strain is both resistant to pyrethroids and carbamates whereas a thorough transcription analysis of this strain using RNAseq clearly indicated that *CYP6P9a/b* were by far the most over-expressed detoxification genes further supporting their cross-resistance role [59].

### 4-Strong association between DNA-based markers of *CYP6P9a/b* and carbamate resistance shows that these PCR-based assays are efficient tools to track cross-resistance

To further understand the role of *CYP6P9a* and *CYP6P9b* in carbamate resistance, the recently available markers of *CYP6P9a/b* were used to carry out a phenotype-genotype correlation study. Genotyping of *CYP6P9a* resistant allele between the alive and dead *An*. *funestus* 24 hours post exposure to bendiocarb and propoxur revealed a strong association between the resistant markers and the ability to survive exposure to bendiocarb and propoxur. This correlation with carbamates is close to what was previously reported for these markers for pyrethroids [22,23].

Previous studies done to assess the combined effect of the 6.5kb SV, *CYP6P9a* and *CYP6P9b* on the efficacy of pyrethroid treated LLINs showed a great increase in survivorship [24]. Results from this study showed that the 6.5kb SV also combines with the *CYP6P9a* resistant genotype to increase the ability of hybrid *An*. *funestus* to survive carbamate exposure. This ability to survive carbamate exposure is more strongly associated with the homozygous resistant for both genes when compared to other genotype combinations. Taking into account the role of the 6.5kb SV enhancer in the increased expression of metabolic resistant genes [24], the 6.5kb SV confers an additive effect in the presence of cis-regulatory promoter factors in *CYP6P9a* and *CYP6P9b* [40]. Hence, the *CYP6P9a*, *CYP6P9b* and the 6.5kb SV P450s PCR markers can be used to investigate any possible cross resistance in *An*. *funestus*. This can help in predicting the impact of known resistance mechanisms on recently introduced insecticide products or those that are under development.

## Conclusion

This study elucidated the underlying molecular bases for carbamate resistance in *An*. *funestus* revealing a major role played by cytochrome P450s notably the duplicated *CYP6P9a* and *-b* in Southern Africa whereas the *CYP6P4a* and *-b* are likely genes of greatest effect in West African populations. Furthermore, *in vivo* and *in vitro* functional analyses demonstrated that *CYP6P9a and -b* metabolise carbamates and that their over-expression is sufficient to confer resistance to this insecticide class as they have previously been shown for pyrethroids. The strong correlation between *CYP6P9a/b* DNA-based resistance markers and carbamate resistance here confirmed that both genes are major drivers of carbamate resistance as they are for pyrethroid resistance. The ability of the DNA-based markers of *CYP6P9a/b* to detect cross-resistance between pyrethroids and carbamates highlights the utility of such tools in detecting and track potential cross-resistance between different insecticide classes. The fact that the same genes can confer resistance to unrelated insecticide classes also represents a challenge for resistance management. Our results highlight the potential danger that complex evolution of resistance to an insecticide class intensely used in the field such as pyrethroids could pose to the efficacy of other insecticides and possibly even to new ones not even yet deployed in the field. Such cross-resistance issues should be taken into account to implement robust insecticide-based intervention using molecular tools available for informed decision making.

## Supporting information

S1 Fig RNAseq transcription profiling of bendiocarb resistance. A) Venn-diagram showing number of differentially expressed genes between different comparisons at FDR<0.05 and Fold-change > 2 (or 1.5 for R-C). B) is for Ghana. R represents bendiocarb resistant *An*. *funestus*, C is for the unexposed *An*. *funestus* and S is FANG.
(TIF)

S2 Fig GO enrichment of the set of transcripts up-regulated in Malawi compared to Uganda. (TIF)

S3 Fig Normalised expression of the top genes over-expressed in Malawi. (A) and Ghana (B) from RNAseq experiments. MWI is Malawi, C is for Control, Perm for Permethrin and Bendio for bendiocarb. C) Correlation between RNAseq and qRT-PCR data combining all data obtained in Ghana and Malawi.
(TIF)

S4 Fig Analysis of list of the significant SNPs between bendiocarb resistant (Southern African) and susceptible (Ugandan) populations. A) Distribution of the different types of variants detected with an enrichment of genes located on the 2R chromosome around the rp1 QTL region harboring *CYP6P9a/b*. B) Quantification of nonsynonymous and synonymous SNPs across the 2R chromosome showing a strong enrichment around the rp1 region further associating this region to bendiocarb resistance.
(TIF)

S5 Fig Susceptibility profile of the hybrid strain between FUMOZ and FANG at F4 used for the genotyping of *CYP6P9a* and the 6.5kb SV markers in relation to carbamate resistance. (TIF)

S6 Fig Correlation between genotypes at *CYP6P9a/6*.*5kb* SV and propoxur resistance. (A) Distribution of 6.5kb SV genotypes between dead and alive mosquitoes after exposure to 0.1% propoxur using WHO bioassay showing that 6.5kb SV significantly allows mosquitoes to survive exposure to this carbamate. (B) Correlation between frequency of 6.5kb SV allele and ability to survive exposure to propoxur. C) Estimation of odds ratio (OR) and associated significance between different genotypes and the ability to survive exposure to propoxur. Ors are given with asterisks indicating level of significance. The arrow within the triangle indicates the direction of OR estimation. For example, Individuals that are RR are 198 times more likely to survive exposure to propoxur that those carrying two copies of the susceptible allele (SS). D), E) and F) are the same respectively for *CYP6P9a*
(TIF)

S1 Text SI methods and SI results. (DOCX)

S1 Table RNASeq alignment metrics for different samples. (XLSX)

S2 Table Transcripts differentially expressed from RNAseq. (XLSX)

S3 Table List of SNPs significant between the resistant Southern African *An*. *funestus* (Malawi and FUMOZ) (R) and bendiocarb susceptible (Uganda and FANG) (S). (XLSX)

S4 Table *An*. *funestus* and flies bioassays results. (XLSX)

S5 Table Resistance marker genotyping in bioassay samples results. (XLSX)
