# Supplementary figures and images for: The duplicated P450s CYP6P9a/b drive carbamates and pyrethroids cross-resistance in the major African malaria vector Anopheles funestus
Source: PLoS Genet. 2023 Mar 27;19(3):e1010678. doi: 10.1371/journal.pgen.1010678 (PMC10089315; doi:10.1371/journal.pgen.1010678)

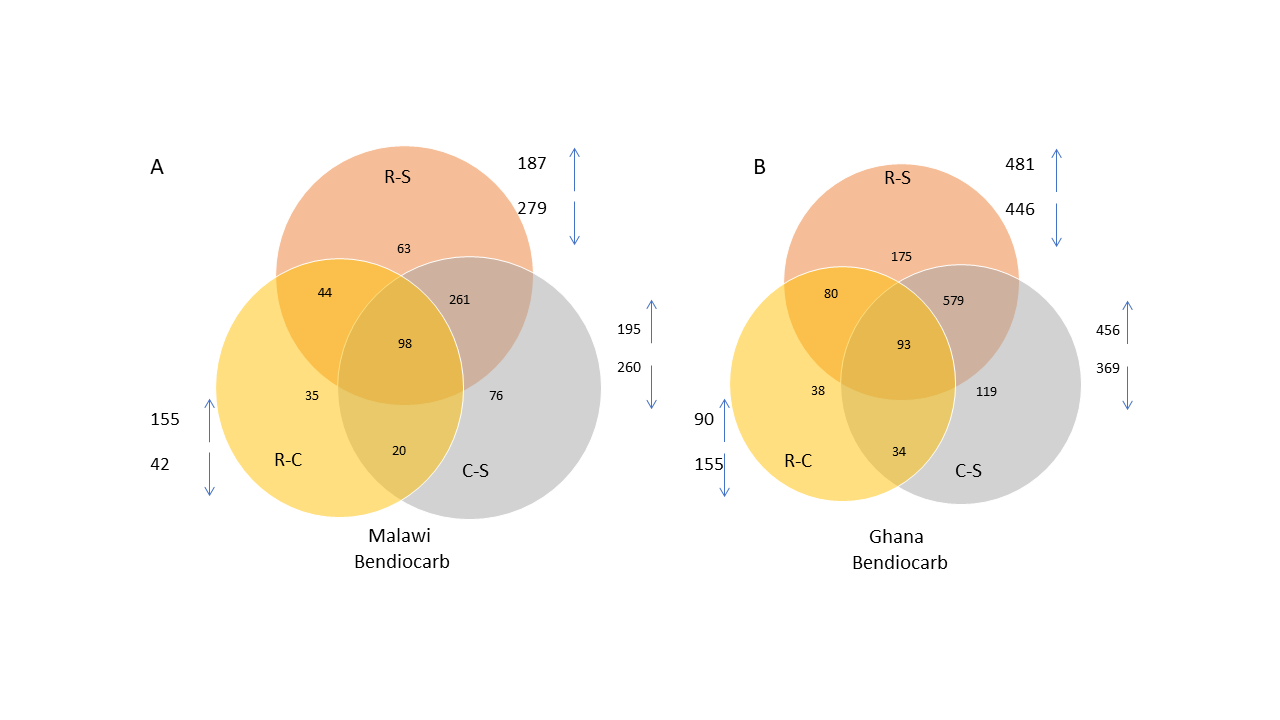

Supplement: S1 Fig — A) Venn-diagram showing number of differentially expressed genes between different comparisons at FDR<0.05 and Fold-change > 2 (or 1.5 for R-C). B) is for Ghana. R represents bendiocarb resistant An. funestus, C is for the unexposed An. funestus and S is FANG. (TIF) [file pgen.1010678.s001.TIF]

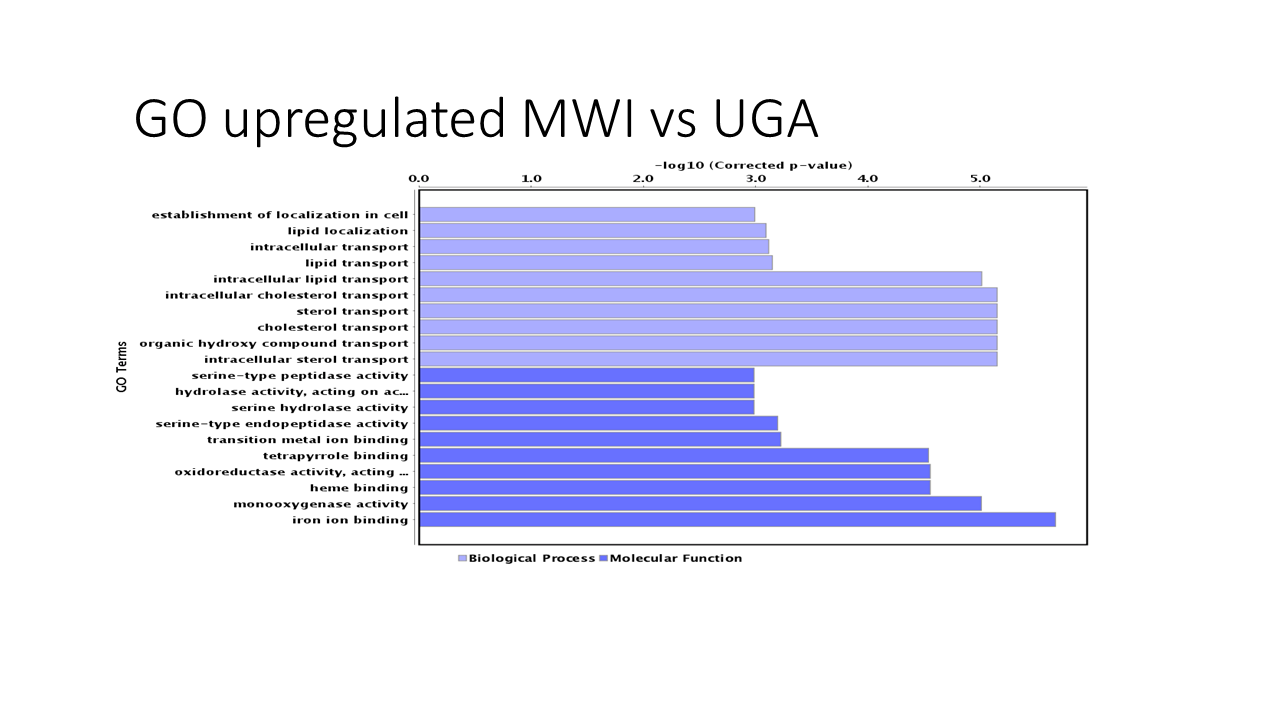

Supplement: S2 Fig — (TIF) [file pgen.1010678.s002.TIF]

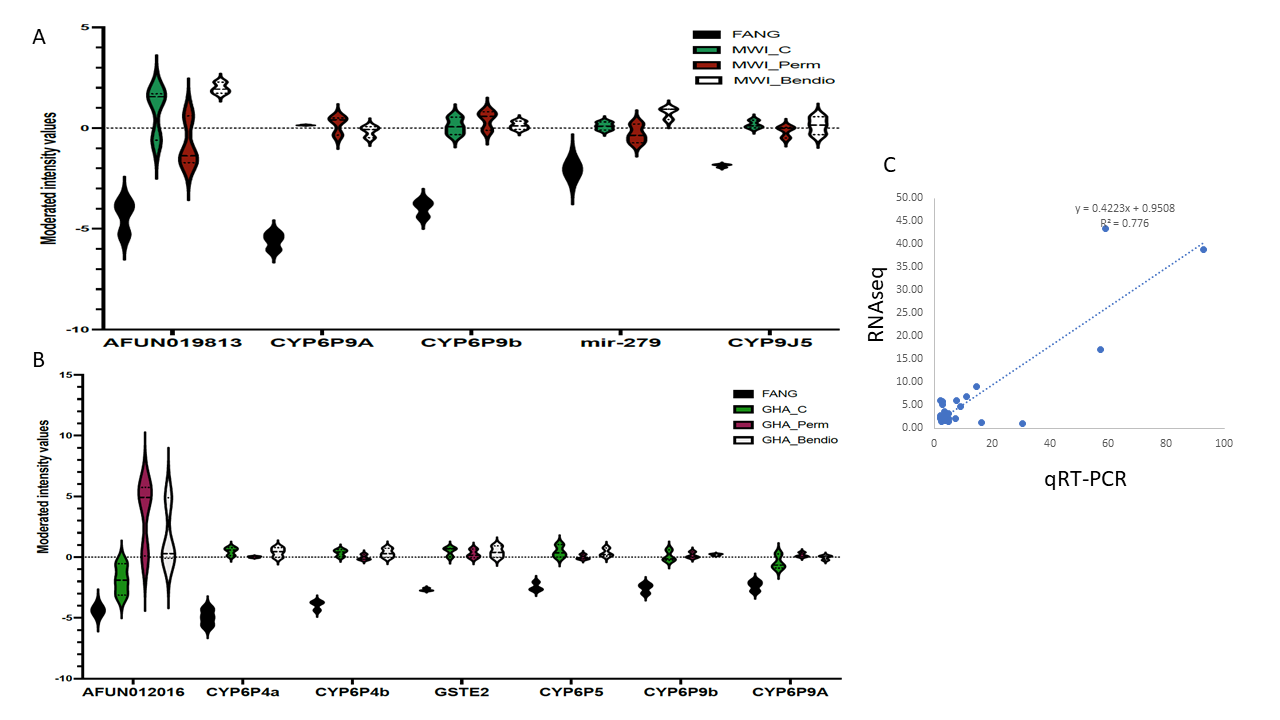

Supplement: S3 Fig — (A) and Ghana (B) from RNAseq experiments. MWI is Malawi, C is for Control, Perm for Permethrin and Bendio for bendiocarb. C) Correlation between RNAseq and qRT-PCR data combining all data obtained in Ghana and Malawi. (TIF) [file pgen.1010678.s003.TIF]

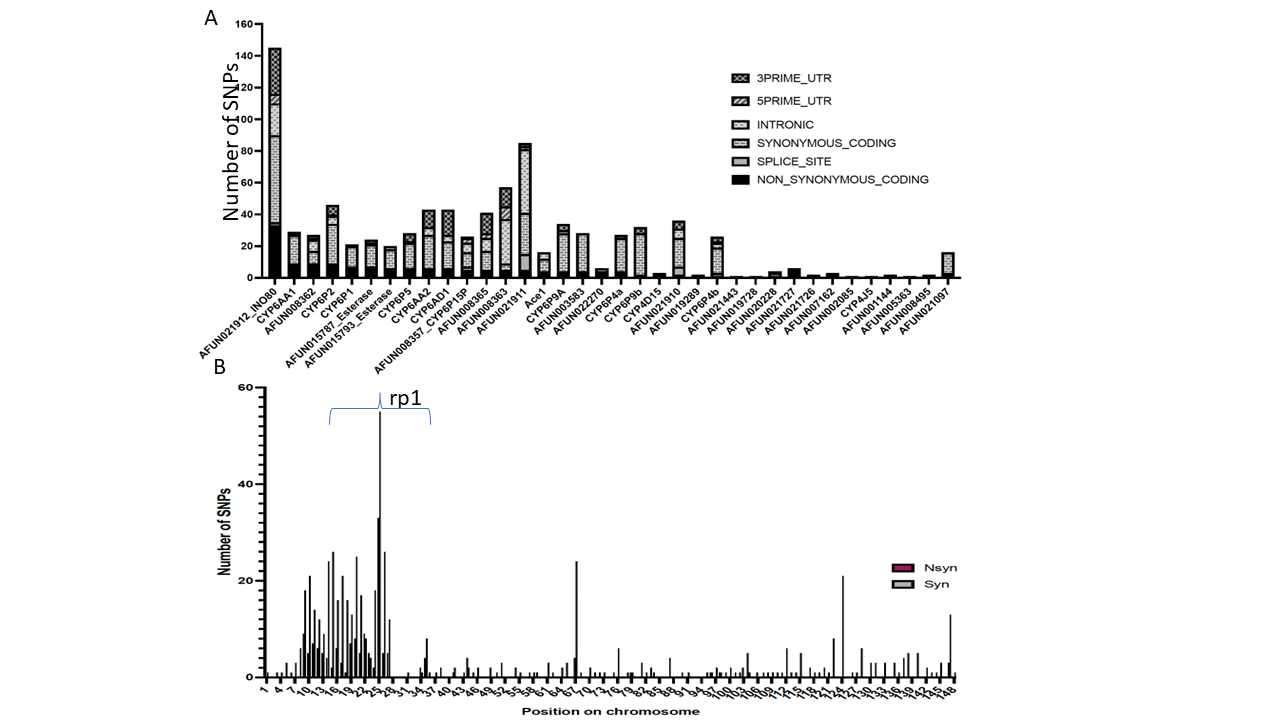

Supplement: S4 Fig — A) Distribution of the different types of variants detected with an enrichment of genes located on the 2R chromosome around the rp1 QTL region harboring CYP6P9a/b. B) Quantification of nonsynonymous and synonymous SNPs across the 2R chromosome showing a strong enrichment around the rp1 region further associating this region to bendiocarb resistance. (TIF) [file pgen.1010678.s004.TIF]

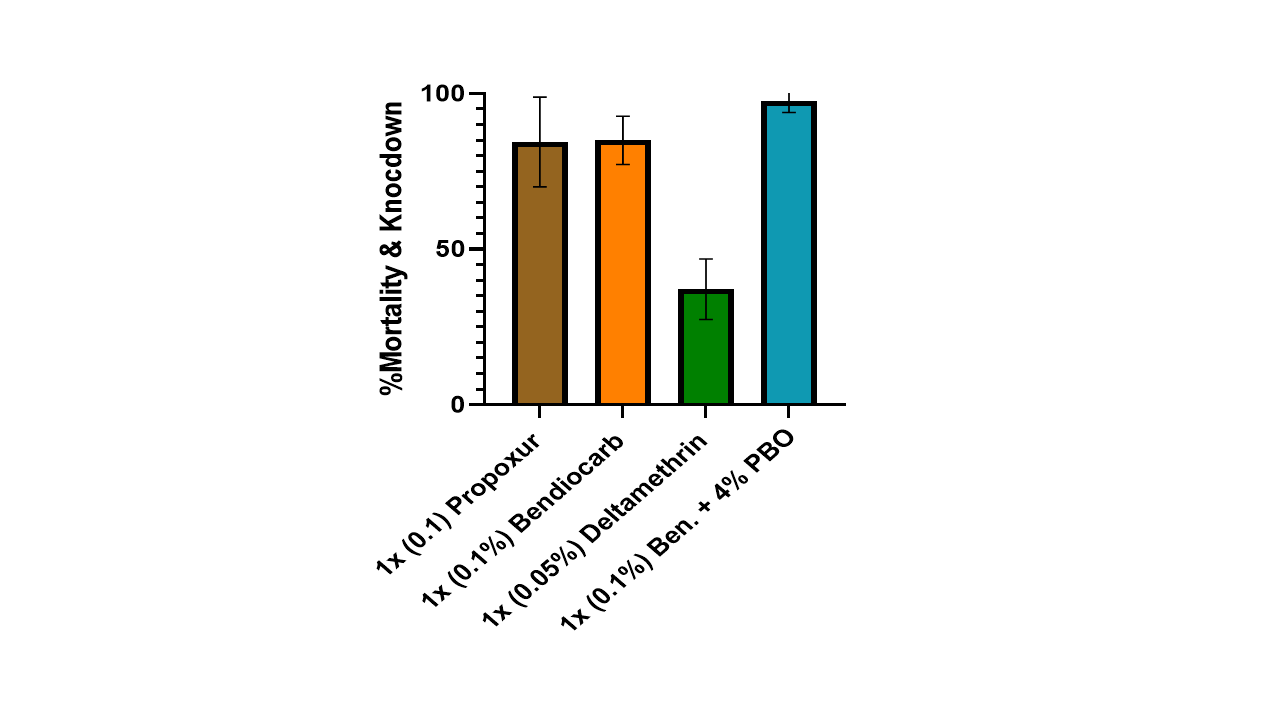

Supplement: S5 Fig — (TIF) [file pgen.1010678.s005.TIF]

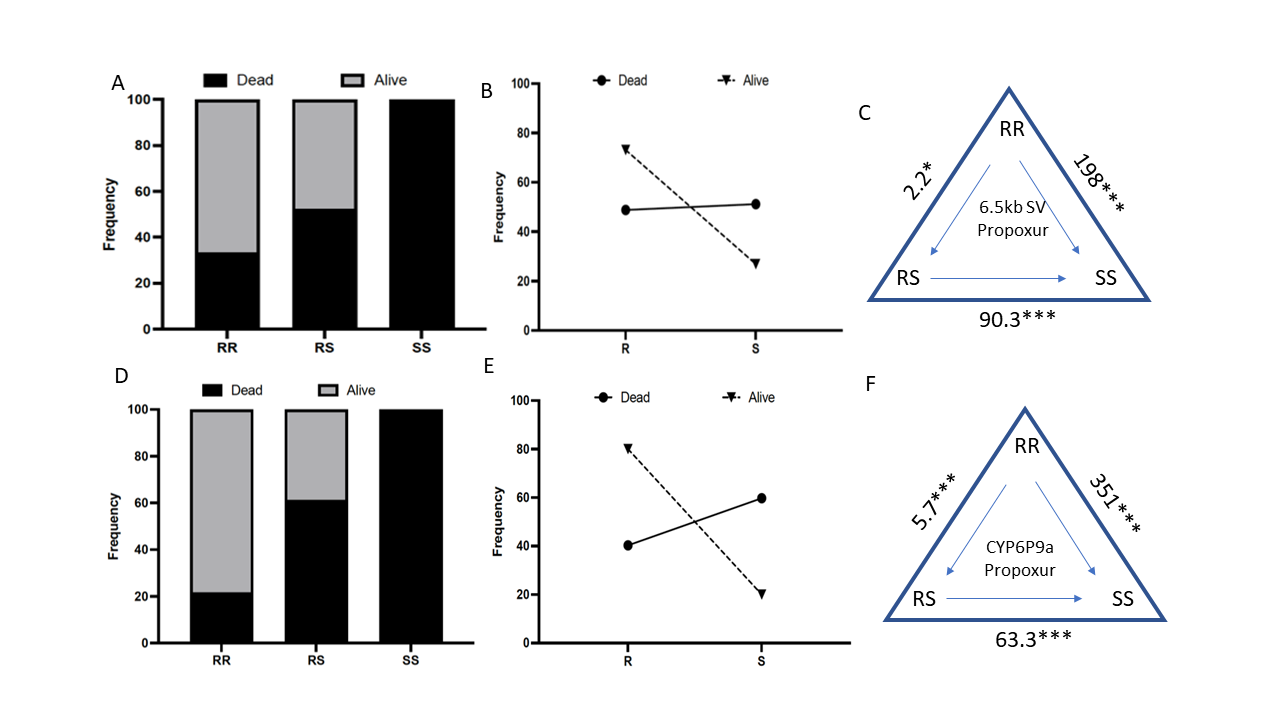

Supplement: S6 Fig — (A) Distribution of 6.5kb SV genotypes between dead and alive mosquitoes after exposure to 0.1% propoxur using WHO bioassay showing that 6.5kb SV significantly allows mosquitoes to survive exposure to this carbamate. (B) Correlation between frequency of 6.5kb SV allele and ability to survive exposure to propoxur. C) Estimation of odds ratio (OR) and associated significance between different genotypes and the ability to survive exposure to propoxur. Ors are given with asterisks indicating level of significance. The arrow within the triangle indicates the direction of OR estimation. For example, Individuals that are RR are 198 times more likely to survive exposure to propoxur that those carrying two copies of the susceptible allele (SS). D), E) and F) are the same respectively for CYP6P9a (TIF) [file pgen.1010678.s006.TIF]
