# Supplementary material for: The duplicated P450s CYP6P9a/b drive carbamates and pyrethroids cross-resistance in the major African malaria vector Anopheles funestus
Source: PLoS Genet. 2023 Mar 27;19(3):e1010678. doi: 10.1371/journal.pgen.1010678 (PMC10089315; doi:10.1371/journal.pgen.1010678)
Supplement: S1 Text — (DOCX) [file pgen.1010678.s007.docx]

**Supporting information**

**Mugenzi et al**

**S1 methodology**

RNAseq and analysis

Briefly, the total RNA was depleted for ribosomal RNA (rRNA) with Ribo-Zero low input kit for human/mouse/rat (Epicentre, Madison, WI, USA) using 100 ng of starting material. The Ribo-Zero mRNA-enriched material was used to prepare RNAseq libraries with the ScriptSeq v2 RNAseq library preparation kit (Epicentre) (15 cycles of PCR amplification). Agencourt AMPure XP beads (Beckman and Coulter, Beverly, MA, USA) were used to purify the libraries followed by a quantification using a Qubit fluorometer (Life Technologies). The size distribution was measured with a 2100 Bioanalyzer (Agilent, Santa Clara, CA, USA).

Illumina adapter sequences (minimum 3 bp match at the 3’ end) were removed from reads by trimming de-multiplexed fastq files using Cutadapt version 1.2.1 [1], and low quality bases were trimmed using Sickle version 1.200 [2] with a minimum window quality score of 20. Reads shorter than 10 bp were removed after trimming. If both reads from a pair passed this filter, each was included in a forward or reverse reads file. If only one of a read pair passed this filter, it was place in an unpaired reads file and not included in analyses.

The analysis used the chromosome-scale *Anopheles funestus* FUMOZ colony reference genome assembly AfunF3 and annotation gene set AfunF3.1 [downloaded from https://www.vectorbase.org/ 25^th^ June 2019 [3]] [4]. To improve the functional annotation of the AfunF3.1 geneset, which contains many putative genes with no descriptions, Blast2Go [5] was used based on the non-redundant (nr) protein database downloaded from NCBI. Blast (BLASTx) searches against the non-redundant (nr) protein database and InterProScan searches of the InterPro protein signature databases were carried out to further annotate the *An. funestus* protein-coding genes.

The Strand NGS software, version 3.4 (Strand Life Sciences, Bangalore, India) was used for data analysis. The AfunF3.1 reference genome was used to align the trimmed R1/R2 read pairs using the option “transcriptome and genome together” (with novel splices) which aligns reads to both transcriptome and genome to find the best matches. Reads that remain unaligned after the first round were further aligned by considering novel splices across known exons and/or novel candidate exons. The best matches for a read were reported if they satisfied the following criteria: a minimum percent identity of 90, a maximum percent gaps between matches of 5, a maximum number of novel splices of 2, a minimum match length of 25, a maximum number of matches to be reported for each read equal to 1 and ignoring reads with matches more than 5 matches. Further quality trimming was performed by removing 3’ends with average base quality less than 10. Raw gene expression was quantified in Strand NGS and raw counts were normalised using DESeq’s inbuilt method [6] which accounts for differences in the total number of reads between samples.

Differential gene expression analysis was performed using DESeq [6] as implemented in Strand NGS. Replicates from different samples were grouped using the “Experiment Setup” section of Strand NGS involving an experiment grouping where replicates of each sample were grouped together and followed by the creation of an experiment indicating the comparisons to be made between different groups. The estimated log_2_ fold change for each transcript was tested using a moderated t-test which is a modification of the unpaired t-test [7]. P-values were adjusted for multiple testing using the False Discovery Rate (FDR) approach of Storey with bootstrapping (q-value). Differentially expressed transcripts were defined as those with an FDR-adjusted P-value < 5% and fold-change >=2. Gene ontology enrichment analysis was subsequently carried out on differentially expressed gene sets using Strand NGS.

**Bait design**

Baits were designed using the SureSelect DNA Advanced Design Wizard in the eArray program of Agilent. The bait size was 120bp for paired-end sequencing using the “centered” option to ensure an even distribution across each target region with the possibility that baits may overlap to regions outside of the target. A bait tiling frequency (indicating the amount of bait overlap) of x3 was used to ensure that even smaller regions in the library could be covered. In total 41082 overlapping RNA probes were generated to cover a total size of 1.931Mbp of the genome (with redundancy) as recently described [8].

**Library construction and capture**: SureSelect library construction and capture were performed by the Centre for Genomic Research (CGR) at the University of Liverpool, using the SureSelect target enrichment custom kit generated with the 41082 probes. The set of 68 samples was used for SureSelect sequence capture and library construction. Libraries were pooled in equimolar amounts and paired-end sequenced (2x150bp) with 20 samples on one run of an Illumina MiSeq, using v4 chemistry. Initial processing and quality control of the targeted sequenced genomic regions were performed using StrandNGS 3.4 (Strand Life Sciences, Bangalore, India) following Hearn, Tagne (9). Alignment and mapping were performed using the “DNA alignment” option against the annotated whole genome of *An. funestus* (version AfunF3.1) [4]. Aligned and mapped reads were used to create an experiment pipeline for DNA variant analysis. A SNP pre-processing quality control step was initially performed before variant detection to reduce false positive calls and involved: (i) a split read re-alignment of partially aligned split reads and noisy normally aligned reads; (ii) local realignment to reduce alignment artifacts around indels; and (iii) base quality score recalibration to reduce errors and systematic bias. The Integrative Genomics Viewer (IGV) [10] was used to visually inspect the alignment results showing that in general, sequence capture regions were well covered and lower level coverage was seen between these regions.

**qRT-PCR**

Total RNA was extracted from three biological replicates from mosquitoes that survived 1 hour of exposure to bendiocarb and permethrin in each country and for FUMOZ and FANG mosquitoes not exposed to any insecticide. One microgram was used as the template for cDNA synthesis using Superscript III (Invitrogen) with oligo-dT20 and RNase H according to the manufacturer’s instructions. The qRT-PCR amplification was performed following standard protocol [11, 12] after establishing the standard curves for each gene to assess PCR efficiency and quantitative differences between samples using serial dilution. For each biological replicate, qRT-PCR was performed with 3 technical replicates. The relative expression level and fold-change (FC) of each target gene were established for comparisons to FANG; these were calculated according to the 2^-ΔΔCT^ method incorporating the PCR efficiency [13] after normalization with the housekeeping genes ribosomal protein S7 (*RSP7*; AFUN007153) and actin 5C (AFUN006819).

**Heterologous Assays**

Expression plasmids pB13::ompA+2-CYP6P9a and pB13::ompA+2*-*CYP6P9b were constructed by fusing cDNA fragment from a bacterial ompA+2 leader sequence with its downstream ala-pro linker to the NH_2_-terminus of the above P450 cDNA, in frame with the P450 initiation codon [14], which was cloned into *Nde*I*-* and *Xba*I-linearised pCW-ori+ vector [15]. Details of PCR conditions for the creation of the expression plasmid cassettes and all primers have already been described in our previous studies [16, 17].

The above plasmids were co-transformed together with *An. gambiae* cytochrome P450 reductase (prepared in a pACYC-184 expression vector) into *E. coli JM109*. Membrane expression was performed as in [14] at 21 °C and 150 rpm, for 40 h after induction with 1 mM IPTG and 0.5 mM δ-ALA to the final concentrations. Membrane content and the P450 reductase activity were determined respectively as previously established [18, 19]. Membrane content and the P450 reductase activity were determined respectively as previously established [18, 19].

**Transgenic expression**

The construction of the transgenic *D. melanogaster* strain was previously described [17, 20]. Briefly, full-length *CYP6P9a and CYP6P9b* were amplified from cDNA using the Phusion High-Fidelity DNA Polymerase (Thermo Scientific) and cloned into the pJET1.2/blunt cloning vector (Thermo Scientific). For each gene, one predominant clone from Malawi was selected and cloned into the pUASattB vector using primers containing *Bgl*II and *Xba*I restriction sites. Using the PhiC31 system, the clones were injected into the germ-line of *D. melanogaster* w^1118^ strain. Two transgenic lines, UAS-CYP6P9a and UAS-CYP6P9b were obtained and balanced. A ubiquitous expression of each transgene in adult F_1_ progeny (experimental group) was obtained after crossing virgin females from the driver strain Act5C-GAL4 ["y [1] w [*]; P(Act5C-GAL4-w) E1/CyO","1;2"] (Bloomington Stock Centre, IN, USA) with UAS-CYP6P9a/b males. Similarly, adult F_1_ control progeny (control group) with the same genetic background as the experimental group but without *CYP6Pa or -b* insert were obtained by crossing virgin females from the driver strain Act5C-GAL4 and UAS recipient line males (which do not carry the pUASattB-CYP6P9a or -b insertion).

## **SI Results:**

## **Transcription profile of bendiocarb resistance**

To detect the set of genes associated with bendiocrb resistance in Malawi, RNAseq was used to compare bendiocarb alive mosquitoes (R) with control (non-exposed, C) (R-C comparison), and to the fully susceptible lab strain, FANG, S (R-S comparison). The control *An. funestus* mosquitoes were also compared to the susceptible FANG strain (C-S). A total of 466 transcripts were differentially expressed between R-S comparison with 187 up-regulated and 279 downregulated in resistant bendiocarb. (Figure S1A). 455 transcripts were differentially expressed when comparing control to FANG (C-S) with 195 upregulated and 260 downregulated in the control versus FANG samples. A lower number of transcripts (197) are differentially expressed in R-C with 155 upregulated in R and 42 downregulated in the resistant versus control samples. A similar approach was taken for Ghana mosquitoes where bendiocarb resistance has also been reported. A total of 927 transcripts were differentially expressed between R-S in Ghana with 481 up-regulated and 446 downregulated. (Figure S1B). 825 transcripts are differentially expressed when comparing control to FANG (C-S) with 456 upregulated and 369 downregulated. 245 were differentially expressed in R-C with 90 upregulated and 155 downregulated.

**References**

1. Martin M. Cutadapt removes adapter sequences from high-throughput sequencing reads. EMBnetjournal. 2011;17:10-2.

2. Joshi NA, Fass JN. Joshi NA, Fass JN. (2011). Sickle: A sliding-window, adaptive, quality-based trimming tool for FastQ files Available at <https://githubcom/najoshi/sickle>. 2011; (Version 1.33) [Software]. .

3. Giraldo-Calderon GI, Emrich SJ, MacCallum RM, Maslen G, Dialynas E, Topalis P, et al. VectorBase: an updated bioinformatics resource for invertebrate vectors and other organisms related with human diseases. Nucleic Acids Res. 2015;43(Database issue):D707-13. doi: 10.1093/nar/gku1117. PubMed PMID: 25510499; PubMed Central PMCID: PMCPMC4383932.

4. Ghurye J, Koren S, Small ST, Redmond S, Howell P, Phillippy AM, et al. A chromosome-scale assembly of the major African malaria vector Anopheles funestus. BioRxiv. 2019;Preprint.

5. Conesa A, Gotz S, Garcia-Gomez JM, Terol J, Talon M, Robles M. Blast2GO: a universal tool for annotation, visualization and analysis in functional genomics research. Bioinformatics. 2005;21(18):3674-6. Epub 2005/08/06. doi: 10.1093/bioinformatics/bti610. PubMed PMID: 16081474.

6. Anders S, Huber W. Differential expression analysis for sequence count data. Genome biology. 2010;11(10):R106. Epub 2010/10/29. doi: 10.1186/gb-2010-11-10-r106. PubMed PMID: 20979621; PubMed Central PMCID: PMCPMC3218662.

7. Smyth GK. Linear models and empirical bayes methods for assessing differential expression in microarray experiments. Stat Appl Genet Mol Biol. 2004;3:Article3. Epub 2006/05/02. doi: 10.2202/1544-6115.1027. PubMed PMID: 16646809.

8. Hearn J, Djoko Tagne CS, Ibrahim SS, Tene-Fossog B, Mugenzi LMJ, Irving H, et al. Multi-omics analysis identifies a CYP9K1 haplotype conferring pyrethroid resistance in the malaria vector Anopheles funestus in East Africa. Mol Ecol. 2022. Epub 2022/05/14. doi: 10.1111/mec.16497. PubMed PMID: 35546741.

9. Hearn J, Tagne CD, Ibrahim SS, Tene-Fossog B, Mugenzi LJ, Irving H, et al. Multi-omics analysis identifies a <em>CYP9K1</em> haplotype conferring pyrethroid resistance in the malaria vector <em>Anopheles funestus</em> in East Africa. bioRxiv. 2021:2021.10.21.465247. doi: 10.1101/2021.10.21.465247.

10. Thorvaldsdottir H, Robinson JT, Mesirov JP. Integrative Genomics Viewer (IGV): high-performance genomics data visualization and exploration. Brief Bioinform. 2013;14(2):178-92. doi: 10.1093/bib/bbs017. PubMed PMID: 22517427; PubMed Central PMCID: PMCPMC3603213.

11. Kwiatkowska RM, Platt N, Poupardin R, Irving H, Dabire RK, Mitchell S, et al. Dissecting the mechanisms responsible for the multiple insecticide resistance phenotype in Anopheles gambiae s.s., M form, from Vallee du Kou, Burkina Faso. Gene. 2013;519(1):98-106. Epub 2013/02/06. doi: 10.1016/j.gene.2013.01.036. PubMed PMID: 23380570; PubMed Central PMCID: PMC3611593.

12. Riveron JM, Irving H, Ndula M, Barnes KG, Ibrahim SS, Paine MJ, et al. Directionally selected cytochrome P450 alleles are driving the spread of pyrethroid resistance in the major malaria vector Anopheles funestus. Proc Natl Acad Sci U S A. 2013;110(1):252-7. doi: 10.1073/pnas.1216705110. PubMed PMID: 23248325; PubMed Central PMCID: PMCPMC3538203.

13. Schmittgen TD, Livak KJ. Analyzing real-time PCR data by the comparative C-T method. Nature Protocols. 2008;3(6):1101-8. doi: 10.1038/nprot.2008.73. PubMed PMID: WOS:000258423700018.

14. Pritchard MP, McLaughlin L, Friedberg T. Establishment of functional human cytochrome P450 monooxygenase systems in Escherichia coli. Methods Mol Biol. 2006;320:19-29. doi: 10.1385/1-59259-998-2:19. PubMed PMID: 16719371.

15. Pritchard MP, Ossetian R, Li DN, Henderson CJ, Burchell B, Wolf CR, et al. A general strategy for the expression of recombinant human cytochrome P450s in Escherichia coli using bacterial signal peptides: expression of CYP3A4, CYP2A6, and CYP2E1. Arch Biochem Biophys. 1997;345(2):342-54. Epub 1997/10/06. doi: S0003-9861(97)90265-4 [pii]

10.1006/abbi.1997.0265. PubMed PMID: 9308909.

16. Ibrahim SS, Riveron JM, Bibby J, Irving H, Yunta C, Paine MJI, et al. Allelic Variation of Cytochrome P450s Drives Resistance to Bednet Insecticides in a Major Malaria Vector. PLoS Genet. 2015;11(10):e1005618. doi: 10.1371/journal.pgen.1005618.

17. Riveron JM, Ibrahim SS, Chanda E, Mzilahowa T, Cuamba N, Irving H, et al. The highly polymorphic CYP6M7 cytochrome P450 gene partners with the directionally selected CYP6P9a and CYP6P9b genes to expand the pyrethroid resistance front in the malaria vector Anopheles funestus in Africa. BMC genomics. 2014;15(1):817. doi: 10.1186/1471-2164-15-817. PubMed PMID: 25261072.

18. Omura T, Sato R. The Carbon Monoxide-Binding Pigment of Liver Microsomes. I. Evidence for Its Hemoprotein Nature. J Biol Chem. 1964;239:2370-8. Epub 1964/07/01. PubMed PMID: 14209971.

19. Strobel HW, Dignam JD. Purification and properties of NADPH-cytochrome P-450 reductase. Methods Enzymol. 1978;52:89-96. Epub 1978/01/01. PubMed PMID: 209290.

20. Riveron JM, Irving H, Ndula M, Barnes KG, Ibrahim SS, Paine MJI, et al. Directionally selected cytochrome P450 alleles are driving the spread of pyrethroid resistance in the major malaria vector Anopheles funestus. P Natl Acad Sci USA. 2013;110(1):252-7. doi: Doi 10.1073/Pnas.1216705110. PubMed PMID: ISI:000313630300059.
